# Supplementary material for: Microbial Degradation of Chromium-Tanned Leather During Thermophilic Composting: A Multi-Scale Analysis of Microbial Communities and Structural Disruption
Source: Biology (Basel). 2025 Dec 18;14(12):1799. doi: 10.3390/biology14121799 (PMC12730970; doi:10.3390/biology14121799)
Supplement: Supplementary file 1 [file biology-14-01799-s001.zip › biology-4012437-supplementary.pdf]

Table S1. Dimensions and weights of leather sample pieces used in small-scale and large-scale composting experiments

SAMPLE	VESSEL	DIMENSIONS (cm)	WEIGHT (g)
1	2L	7 x 1.5	1.39
2	2L	7 x 1.5	1.42
3	2L	7 x 1.5	1.25
4	40L	4.8 x 10.5	6.92
5	40L	5.5 x 10	6.99
6	40L	5.5 x 10	6.95

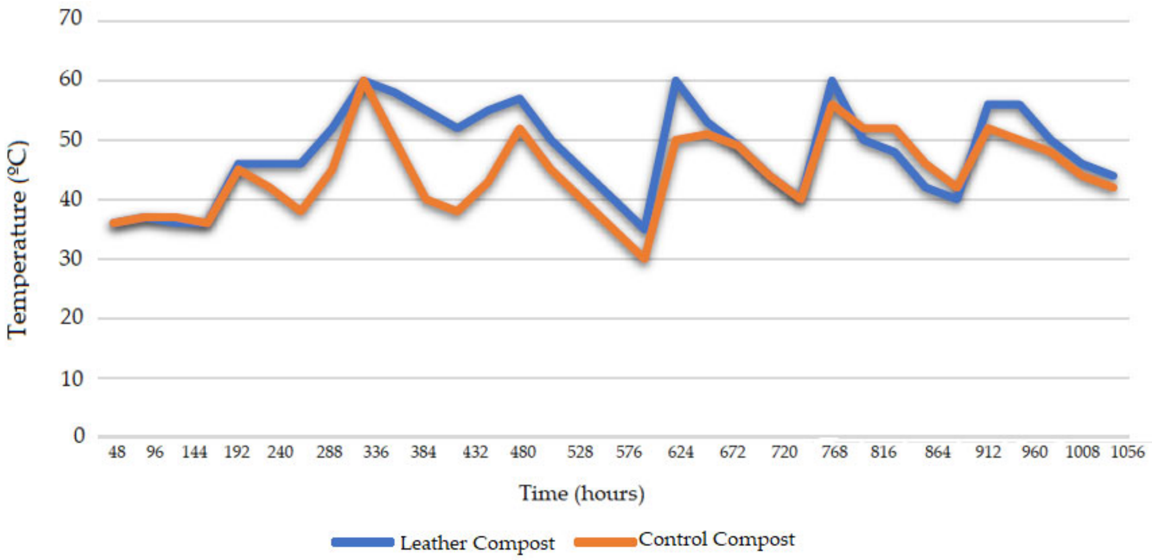

Figure S1. Temperature profile of the 2 L Dewar composting system over 1056 hours. Thermophilic conditions (≥50 °C) were rapidly achieved but exhibited frequent fluctuations. Reinoculations (n = 5) were performed when temperature dropped below 50 °C, approximately at hours 160, 288, 576, 720 and 864.

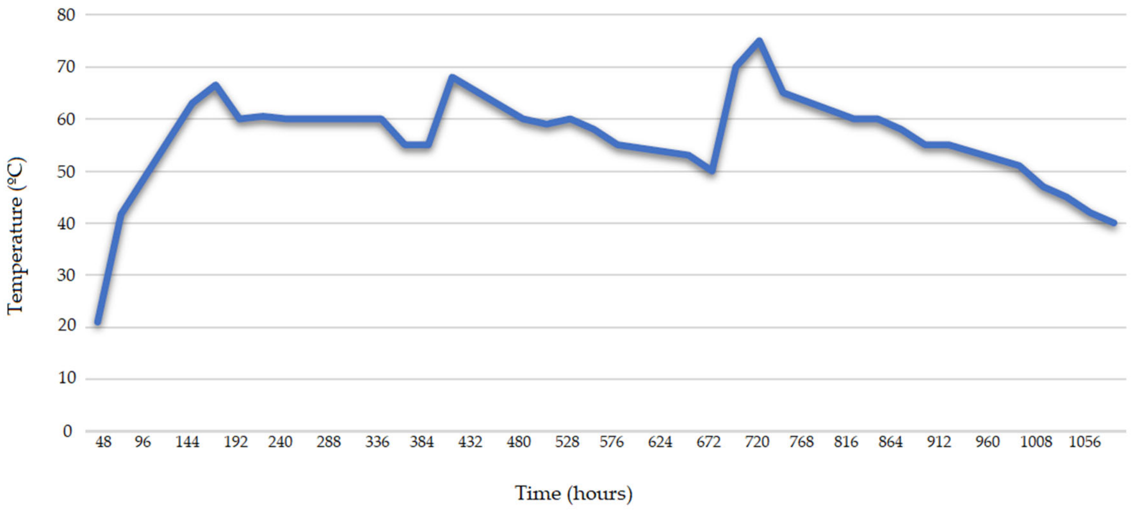

Figure S2. Temperature profile of the 40 L Dewar composting system over 1056 hours. Sustained thermophilic conditions were observed, with peak temperatures exceeding 70 °C. Reinoculations (n = 2) were carried out when temperature dropped below 55 °C, at approximately hours 384 and 672.

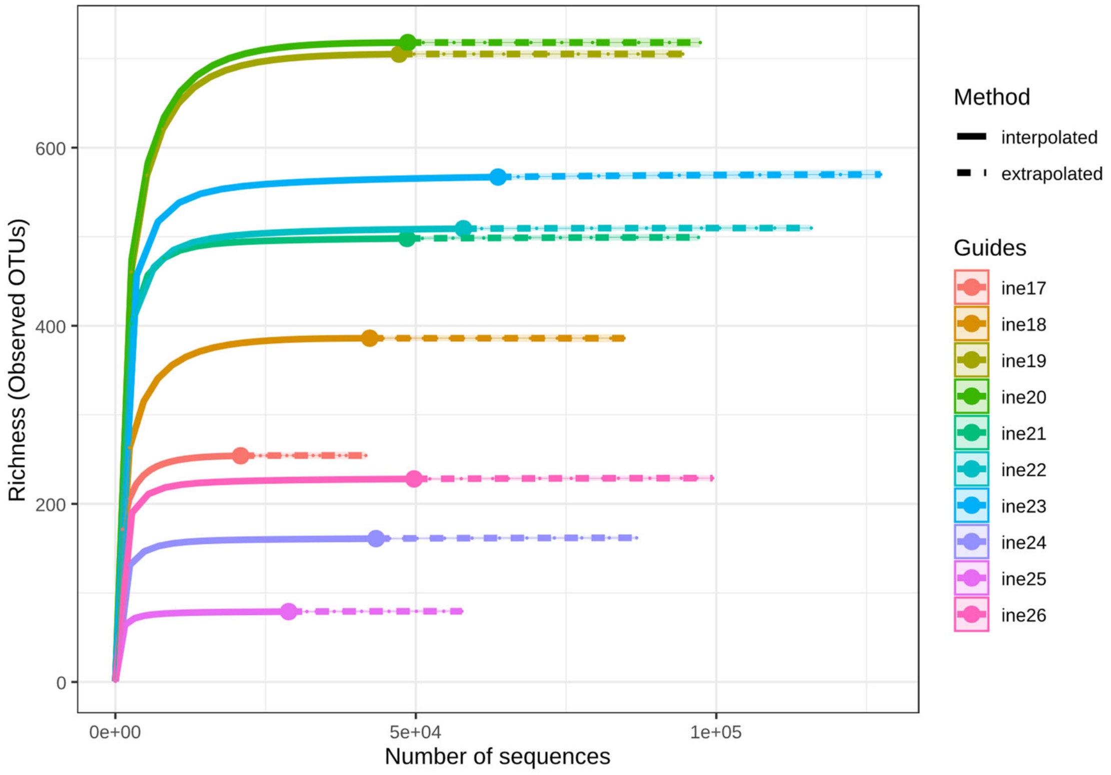

Figure S3. Rarefaction curves based on OTU richness for all compost samples. Curves are shown as interpolated (solid lines) and extrapolated (dashed lines), indicating sequencing depth was sufficient to capture the microbial diversity of each sample. Sample identifiers (ine17–ine26) correspond to samples from 2 L and 40 L Dewar vessels (Table 2).

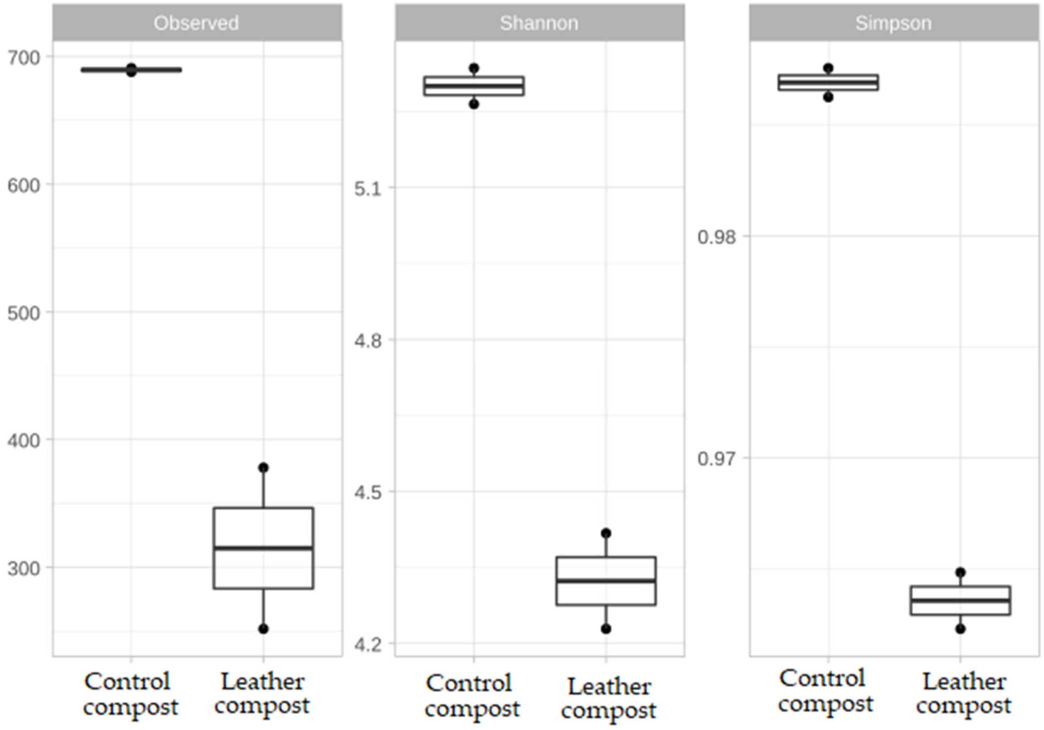

Figure S4. Alpha diversity metrics (Observed richness, Shannon index, Simpson index) at ASV level for 2 L Dewar vessel samples (ine17–ine20) grouped by sample type. Control compost samples show higher diversity compared to leather compost samples.

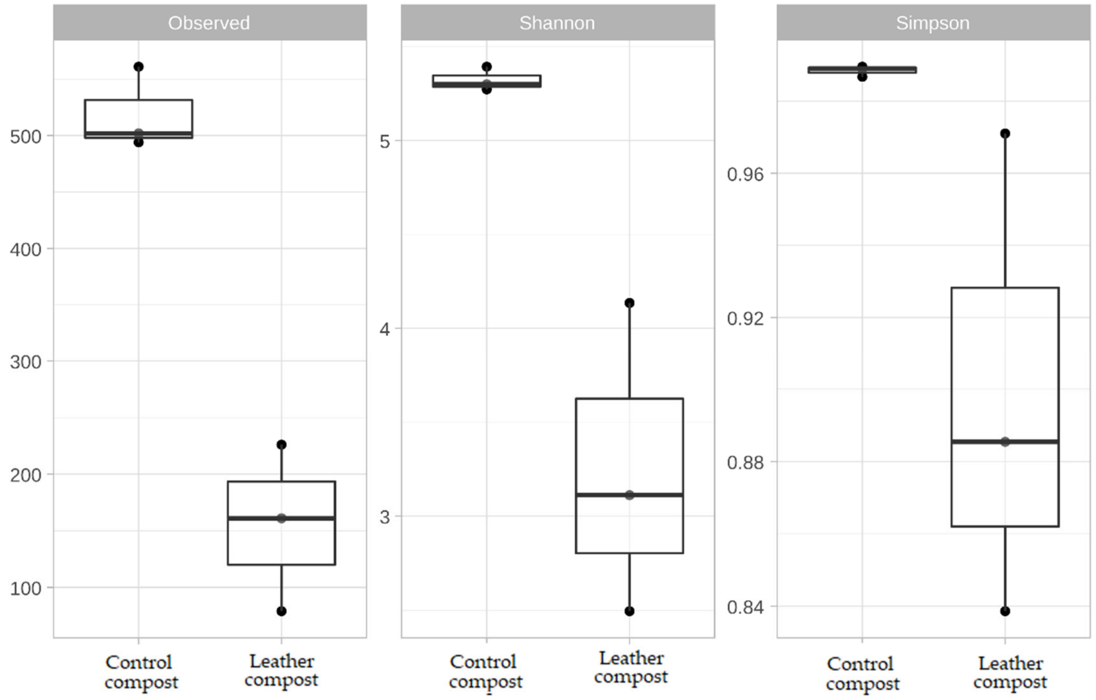

Figure S5. Alpha diversity metrics (Observed richness, Shannon index, Simpson index) at ASV level for 40 L Dewar vessel samples (ine21–ine26)) grouped by sample type. Control compost samples show higher diversity compared to leather compost samples.

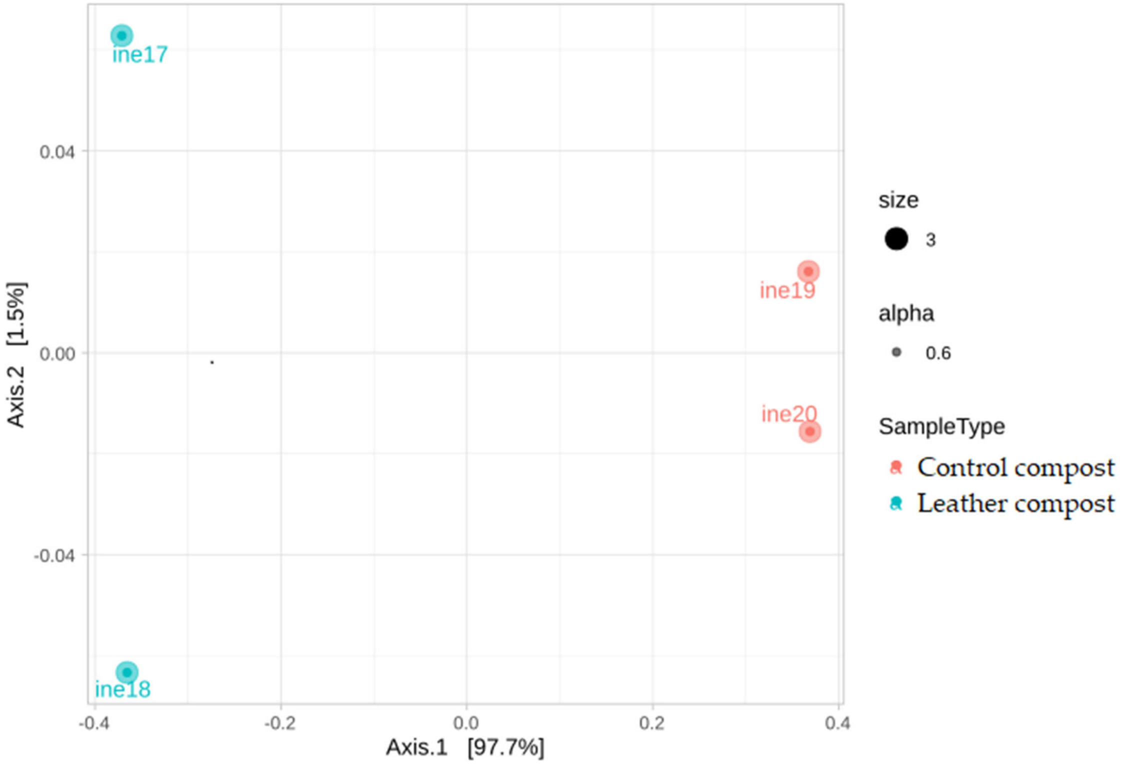

Figure S6. Principal Coordinates Analysis (PCoA) of bacterial β-diversity based on Bray–Curtis dissimilarity for small-scale composting system samples (ine17–ine20). The percentage shown on Axis 1 represents the variance explained by that axis alone. Control and leather compost samples from two distinct clusters, indicating compositional differences in microbial communities.

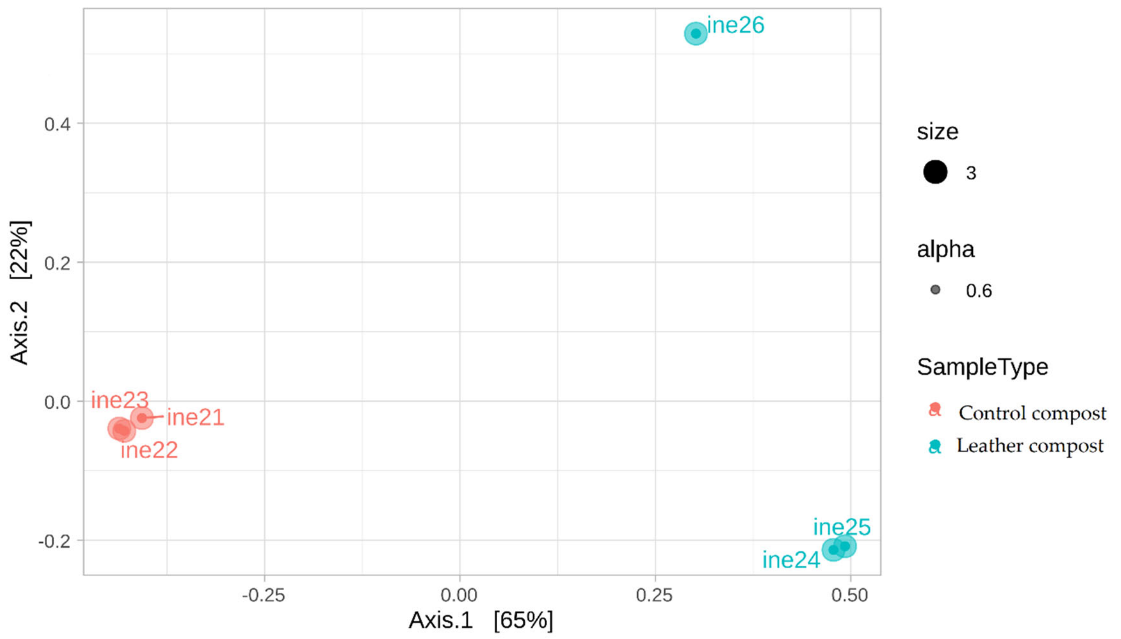

Figure S7. Principal Coordinates Analysis (PCoA) of bacterial β-diversity based on Bray–Curtis dissimilarity for large-scale composting system samples (ine21–ine26). The percentage shown on Axis 1 represents the variance explained by that axis alone. Control and leather compost samples cluster separately, indicating consistent differences in microbial community composition across treatments.

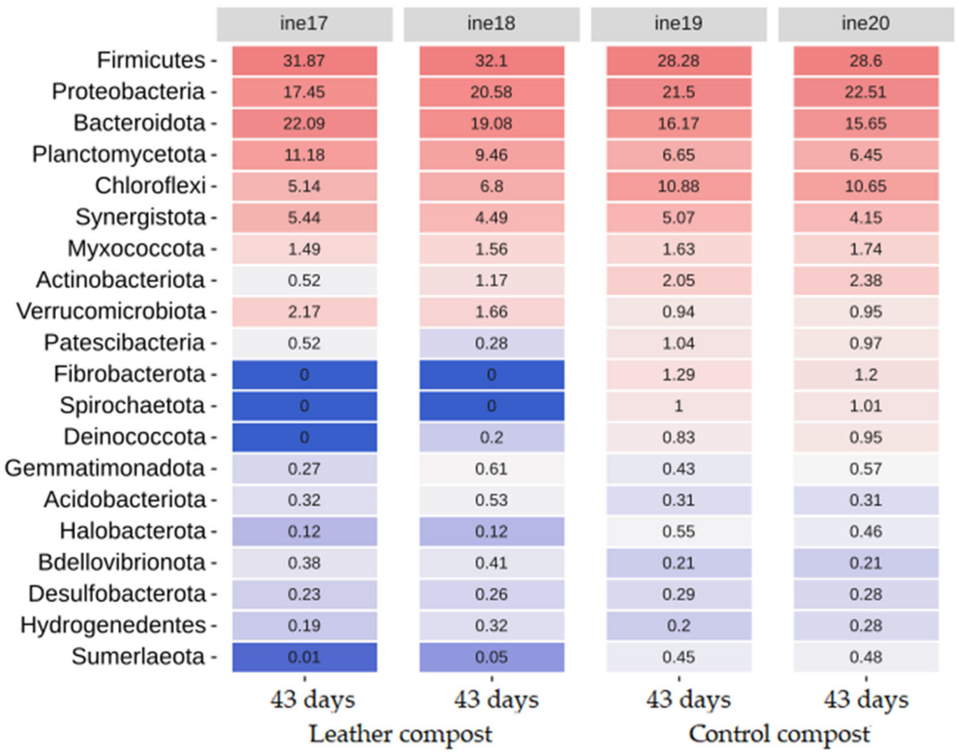

Figure S8. Relative abundances of bacterial phyla in samples ine17–ine20 (2 L Dewar vessel).. Values are expressed as proportions of total ASVs per sample.

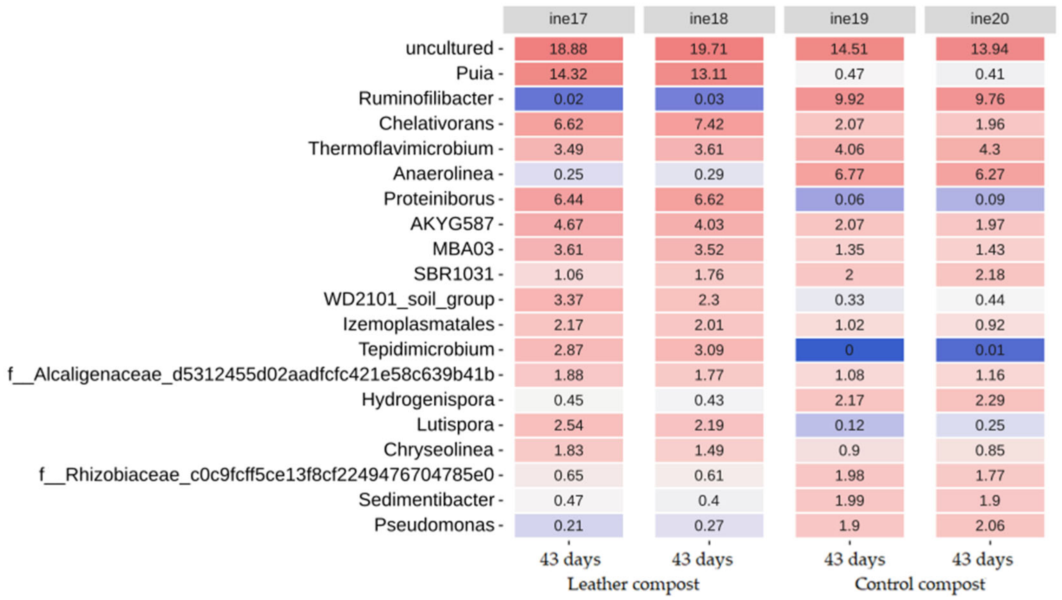

Figure S9. Relative abundances of bacterial genera in samples ine17–ine20 (2 L Dewar vessel). "Uncultured" category aggregates all unclassified or uncultured ASVs at the genus level.

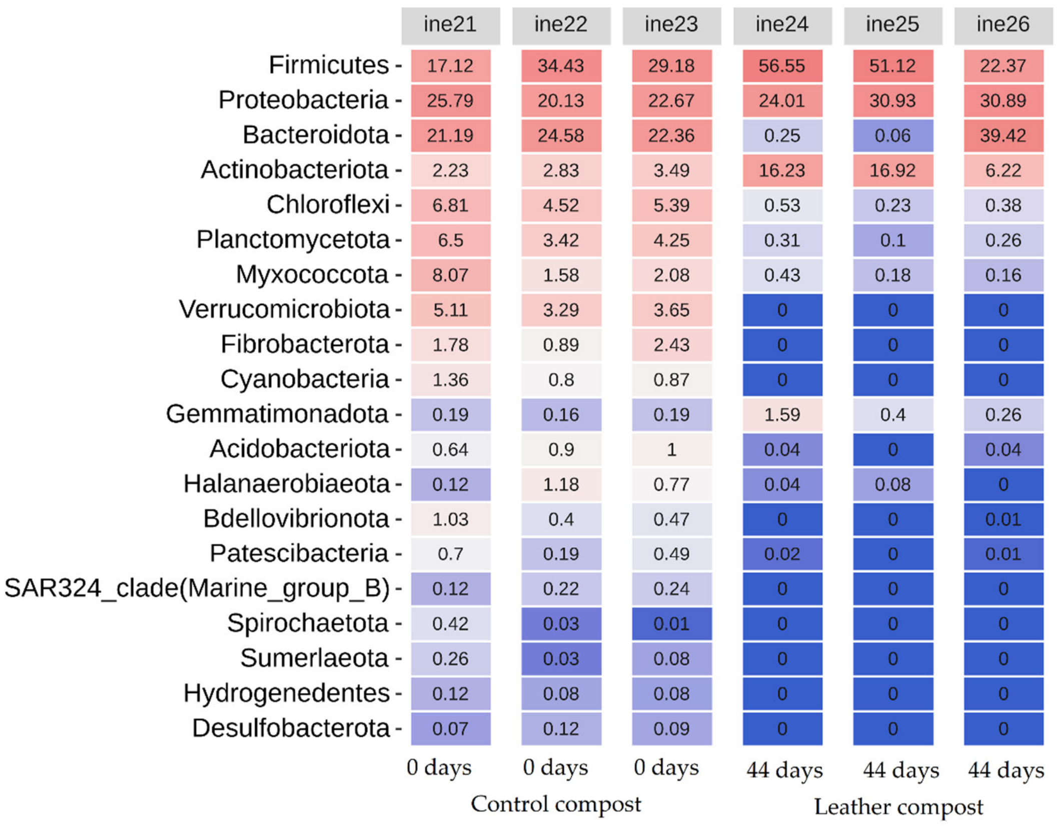

Figure S10. Relative abundances of bacterial phyla in samples ine21–ine26 (40 L Dewar vessel). Values are expressed as proportions of total ASVs per sample.

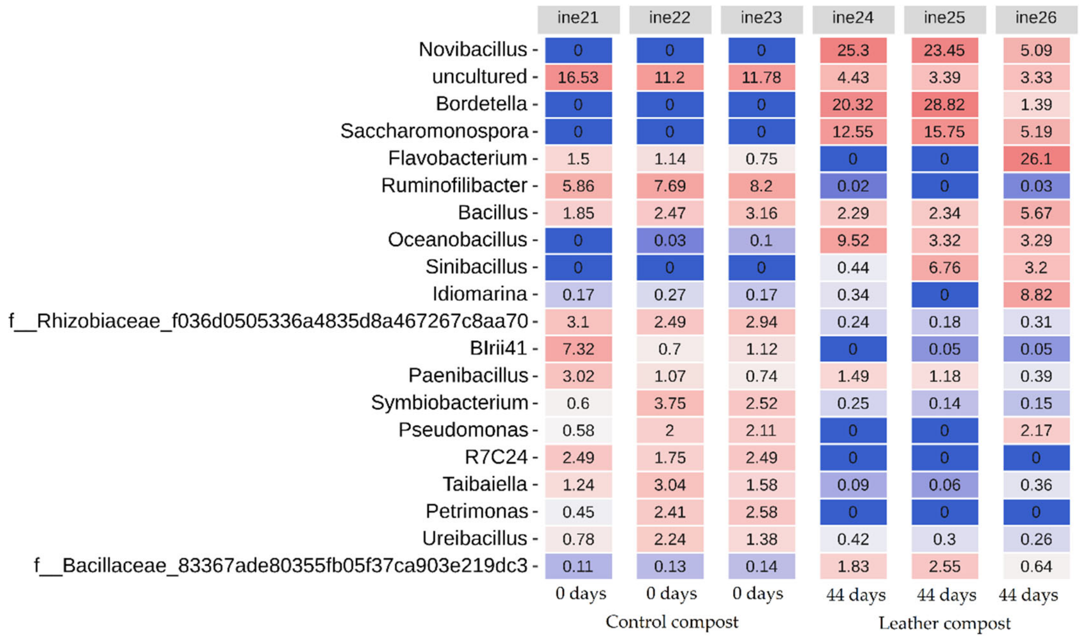

Figure S11. Relative abundances of bacterial genera in samples ine21–ine26 (40 L Dewar vessel). "Uncultured" category aggregates all unclassified or uncultured ASVs at the genus level.

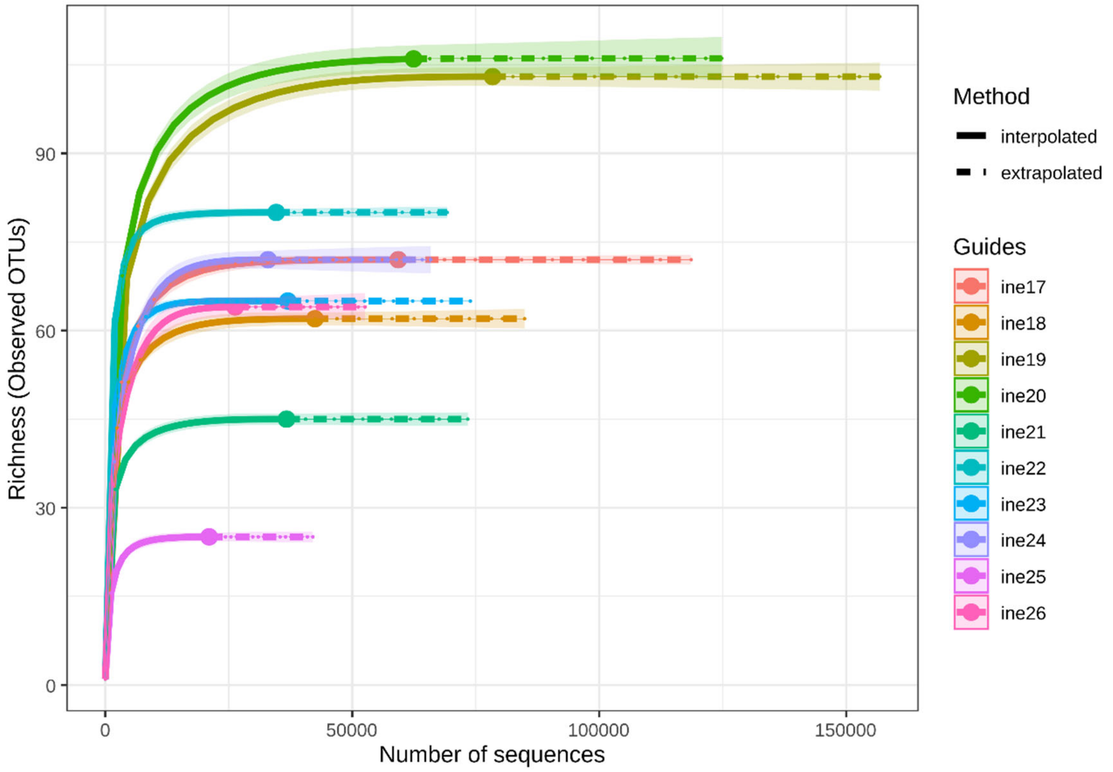

Figure S12. Rarefaction curves at the OTU level for fungal samples ine17–ine26. Solid lines indicate interpolated richness; dashed lines indicate extrapolated richness. All curves reached saturation, confirming adequate sequencing depth. Sample identifiers (ine17–ine26) correspond to samples from 2 L and 40 L Dewar vessels (Table 2).

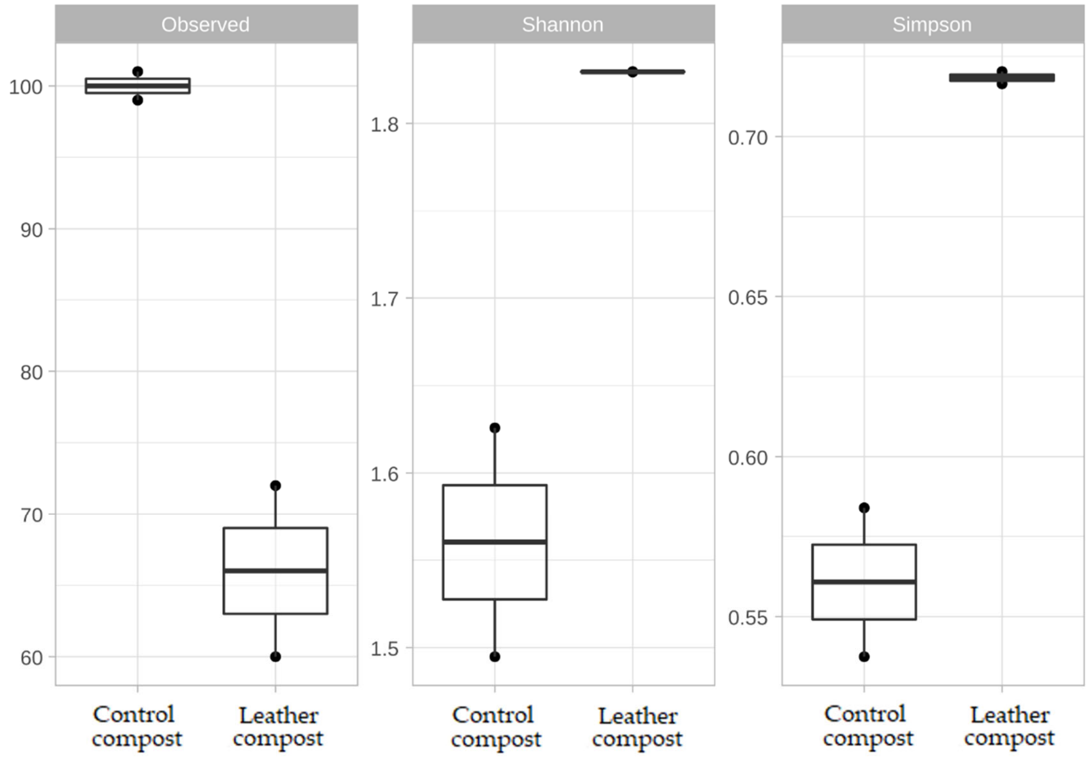

Figure S13. Alpha diversity (Observed richness, Shannon index, Simpson index) at ASV level for fungal samples ine17–ine20 (2 L Dewar vessel).

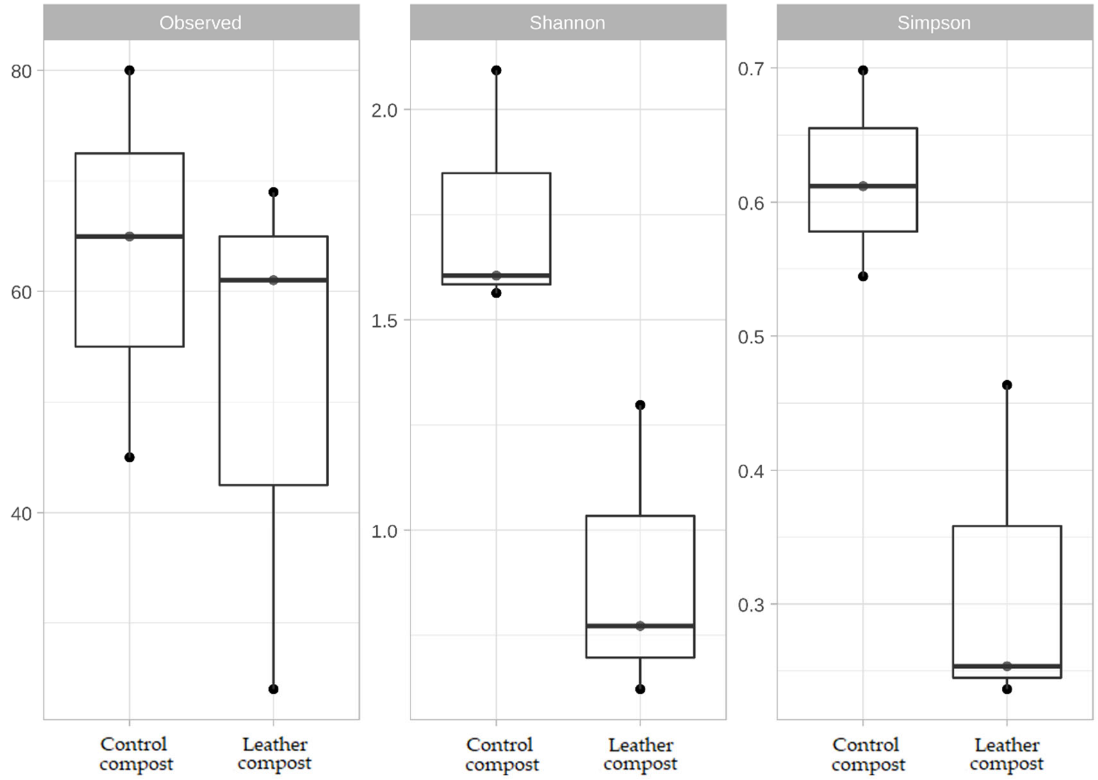

Figure S14. Alpha diversity (Observed richness, Shannon index, Simpson index) at ASV level for fungal samples ine21–ine26 (40 L Dewar vessel).

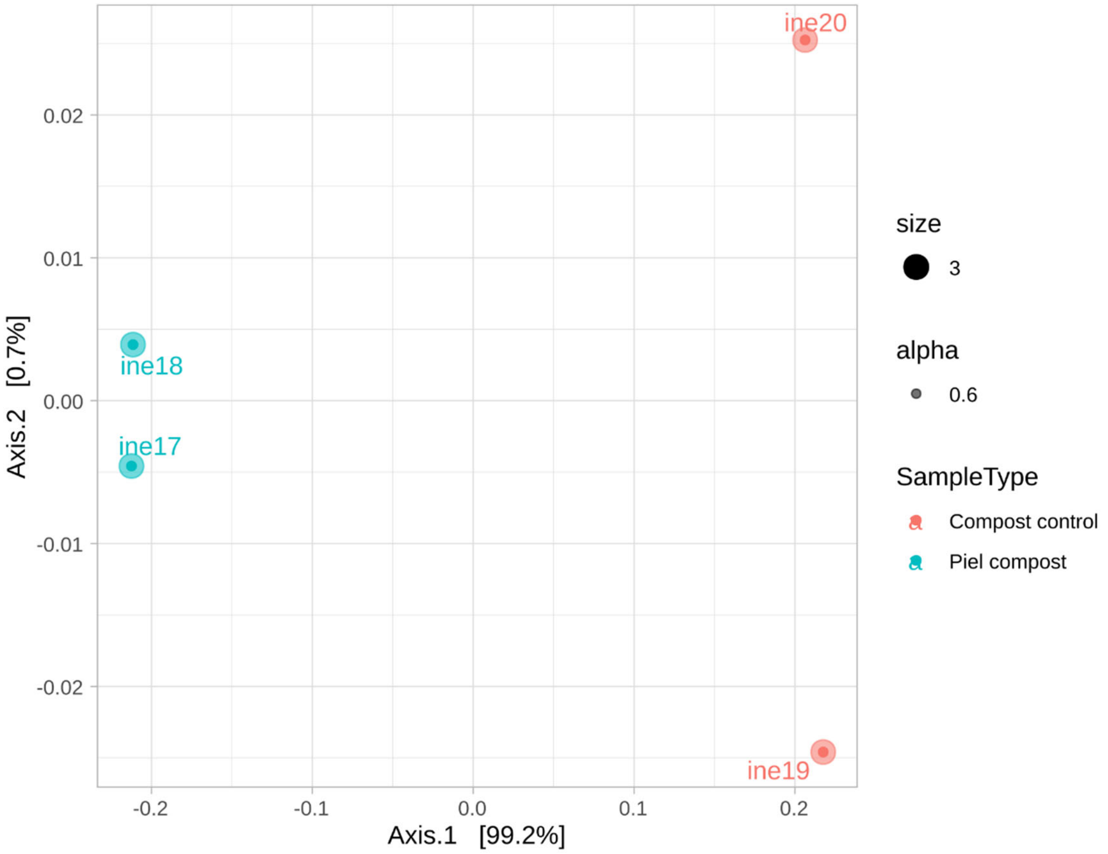

Figure S15. Principal Coordinates Analysis (PCoA) of fungal β-diversity based on Bray–Curtis dissimilarity for small-scale composting system samples (ine17–ine20). The percentage shown on Axis 1 represents the variance explained by that axis alone.

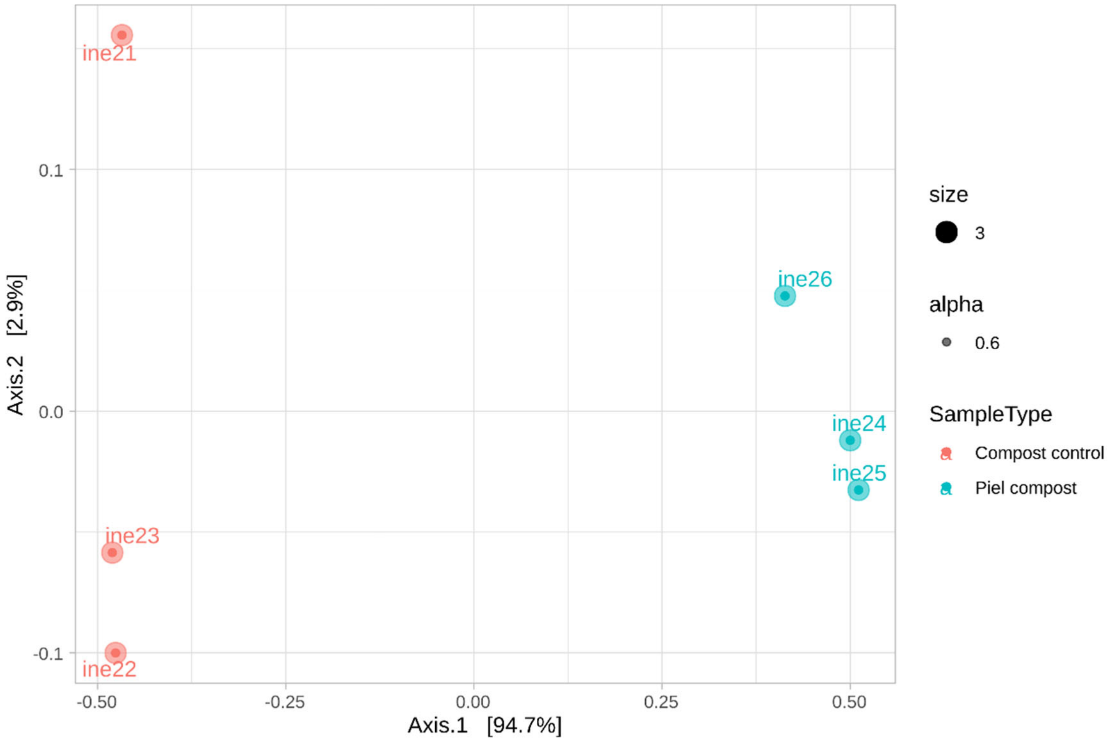

Figure S16. Principal Coordinates Analysis (PCoA) of fungal β-diversity based on Bray–Curtis dissimilarity for large-scale composting system samples (ine21–ine26). The percentage shown on Axis 1 represents the variance explained by that axis alone.

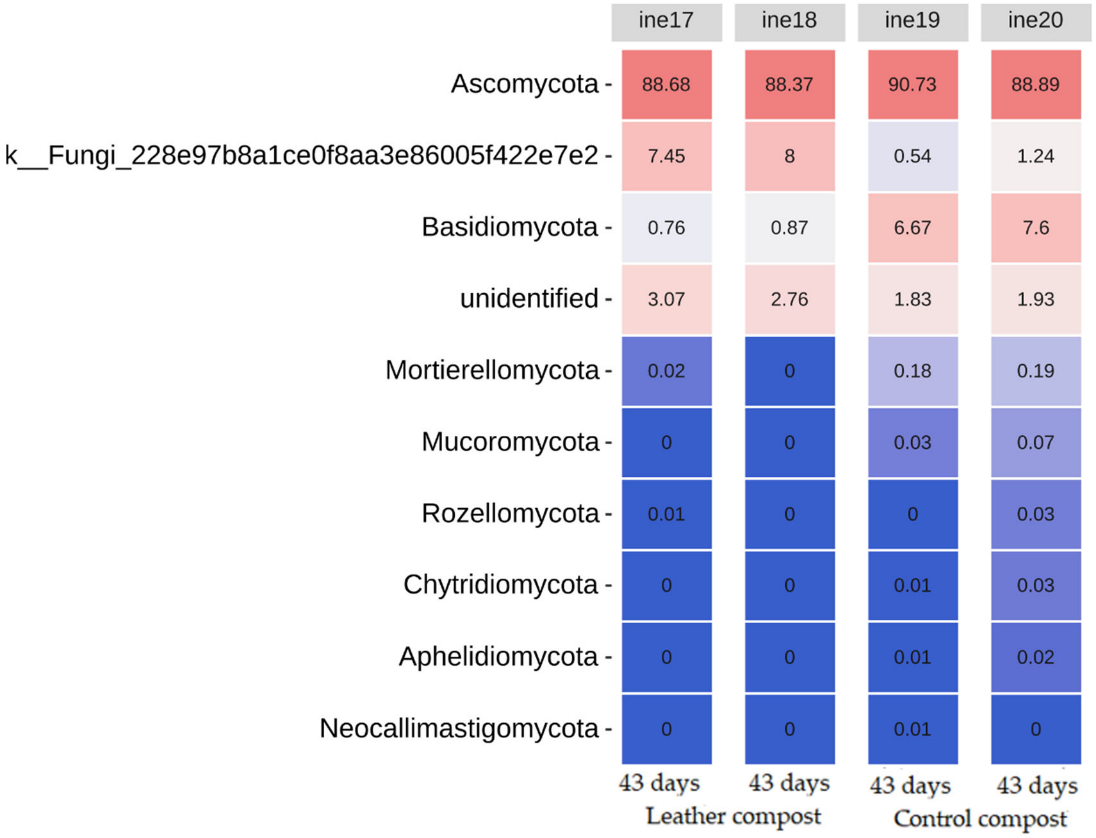

Figure S17. Relative abundances of fungal phyla in samples ine17–ine20 (2 L Dewar vessel). Expressed as percentage of total ASVs per sample.

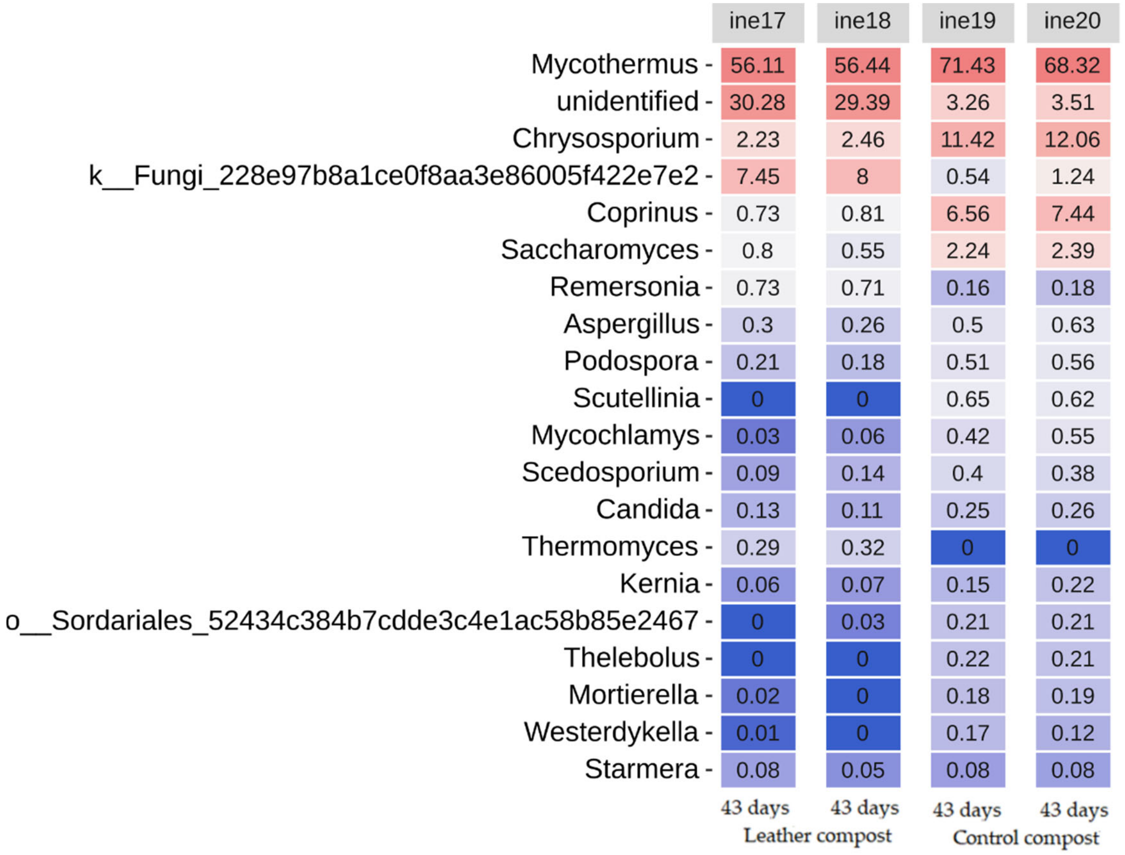

Figure S18. Relative abundances of fungal genera in samples ine17–ine20 (2 L Dewar vessel). The "uncultured" category includes unclassified ASVs at the genus level.

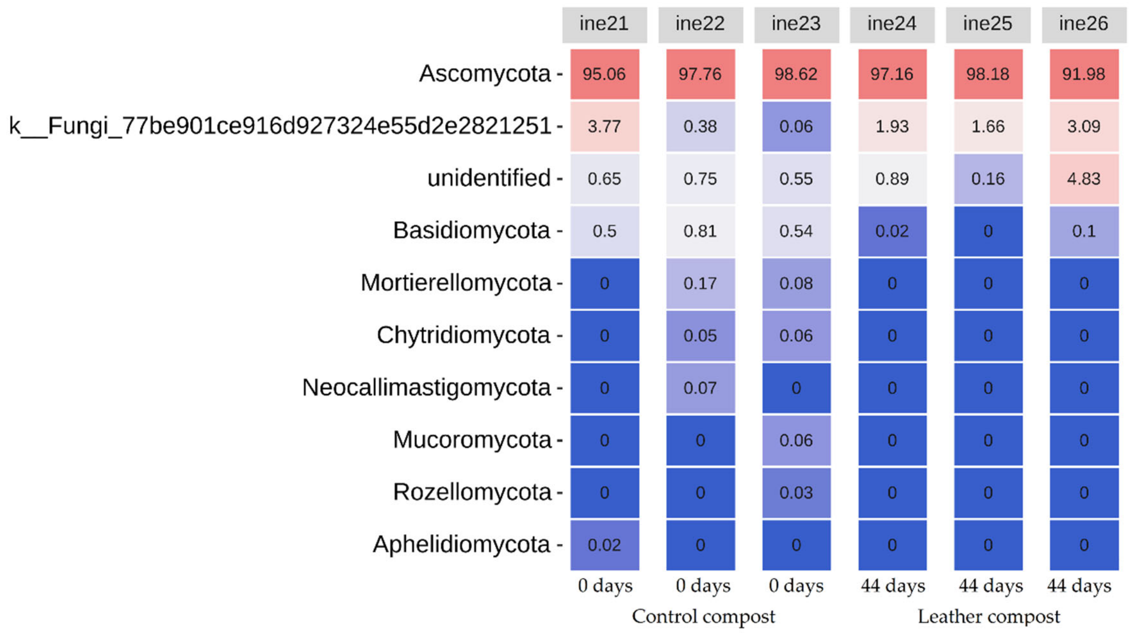

Figure S19. Relative abundances of fungal phyla in samples ine21–ine26 (40 L Dewar vessel). Expressed as percentage of total ASVs per sample.

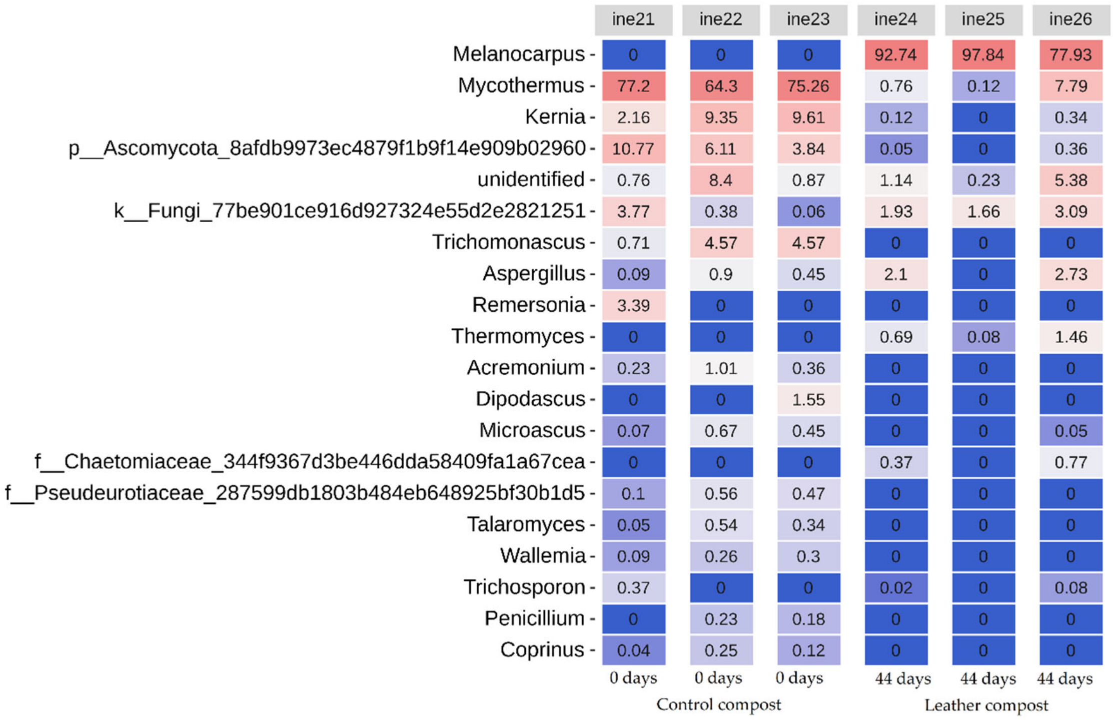

Figure S20. Relative abundances of fungal genera in samples ine21–ine26 (40 L Dewar vessel). The "uncultured" category includes unclassified ASVs at the genus level.

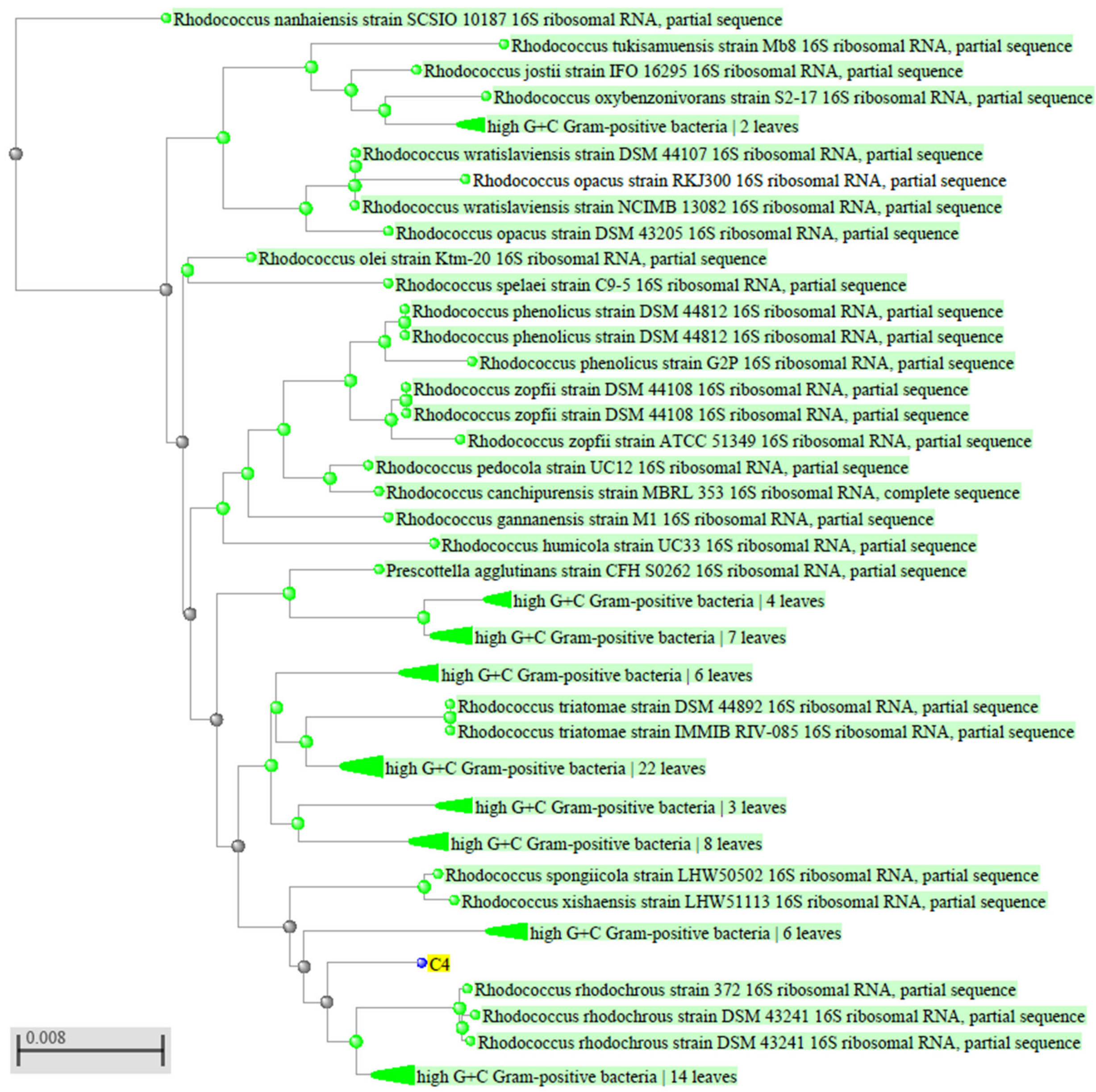

Figure S21. Phylogenetic tree of strain C4 based on 16S rRNA gene sequences showing its relationship to *Rhodococcus rhodochrous* strain DSM 43241.

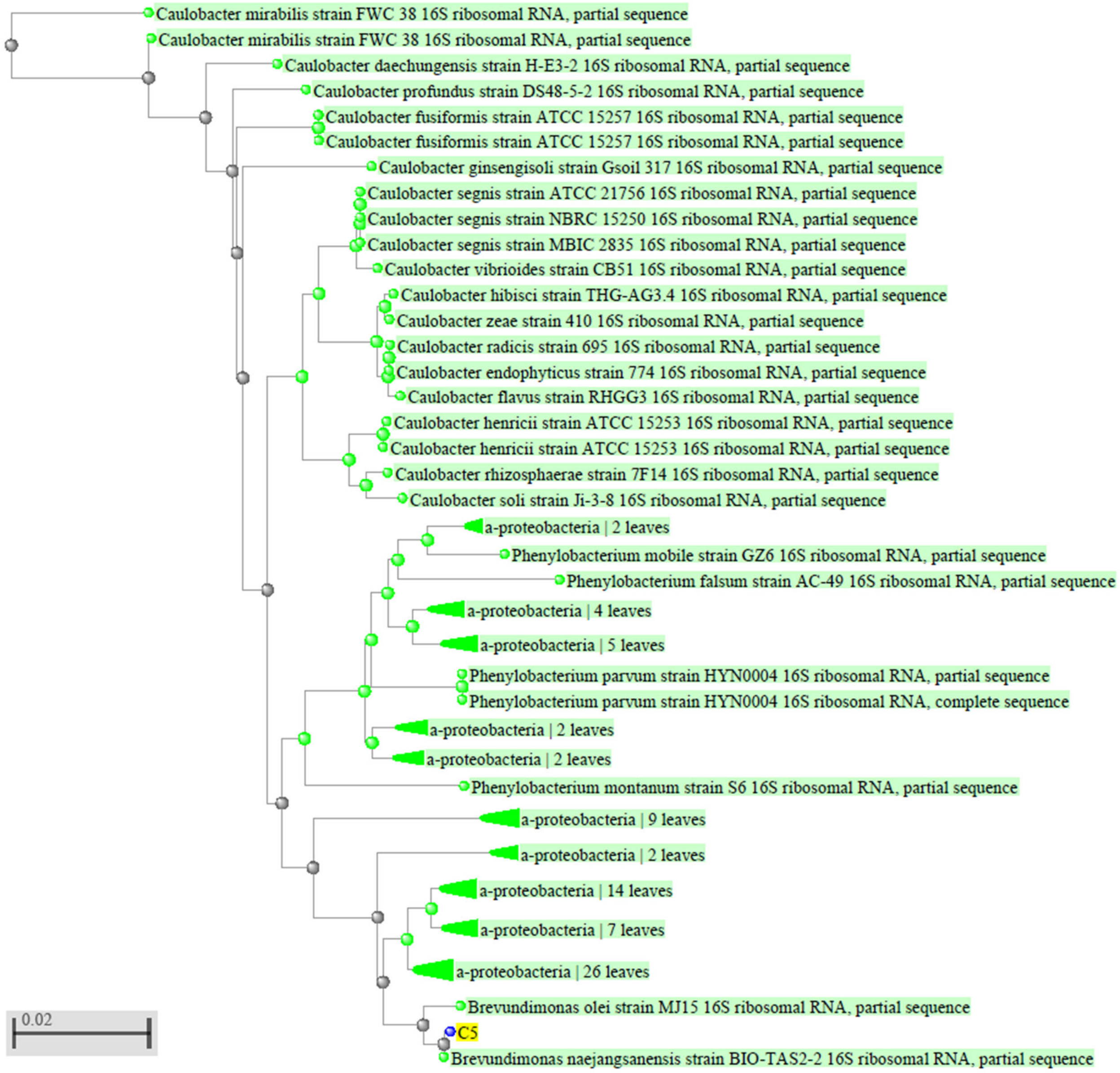

Figure S22. Phylogenetic tree of strain C5 based on 16S rRNA gene sequences showing its relationship to *Brevundimonas naejangsanensis* strain BIO-TAS2-2.

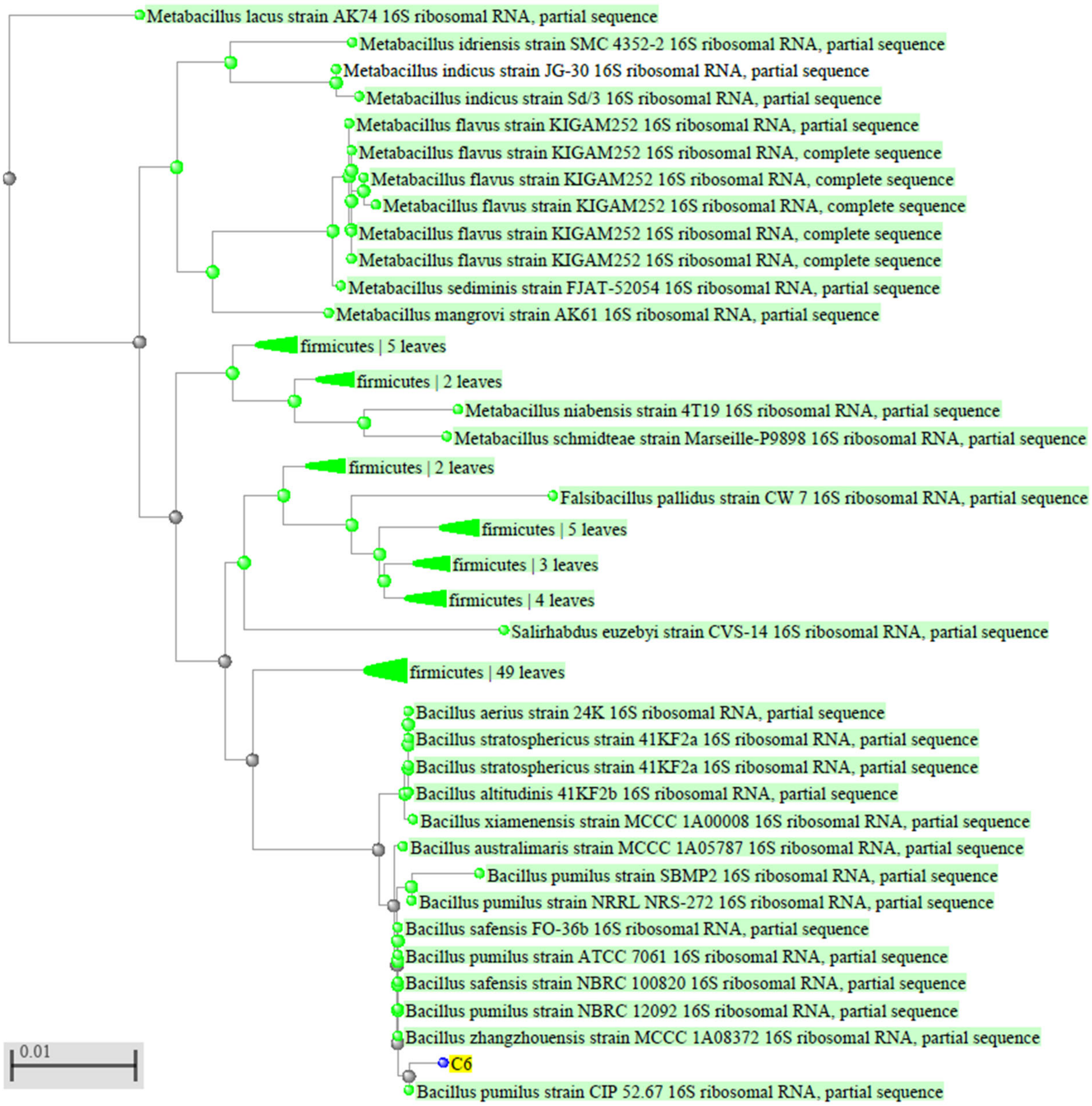

Figure S23. Phylogenetic tree of strain C6 based on 16S rRNA gene sequences showing its relationship to *Bacillus zhangzhouensis* strain MCCC 1A08372.

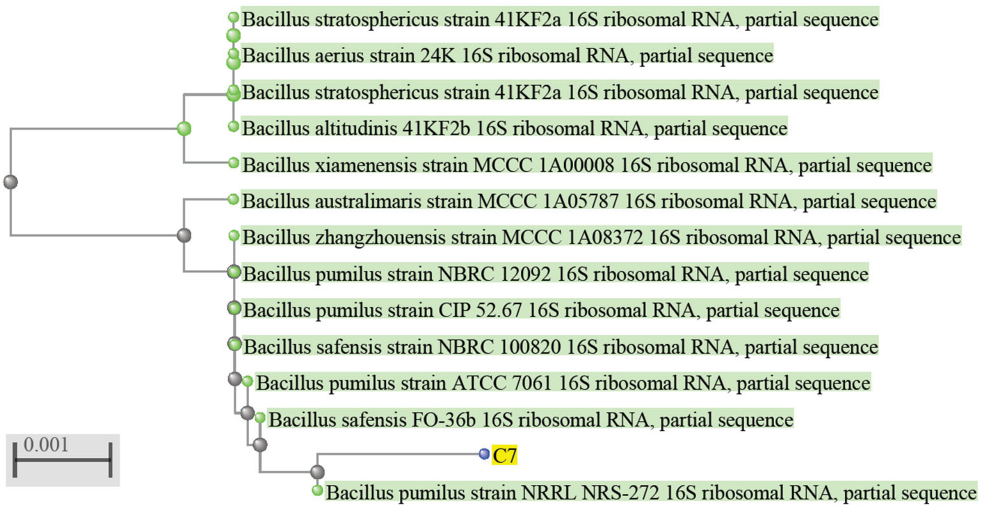

Figure S24. Phylogenetic tree of strain C7 based on 16S rRNA gene sequences showing its relationship to *Bacillus pumilus* strain NBRC 12092.

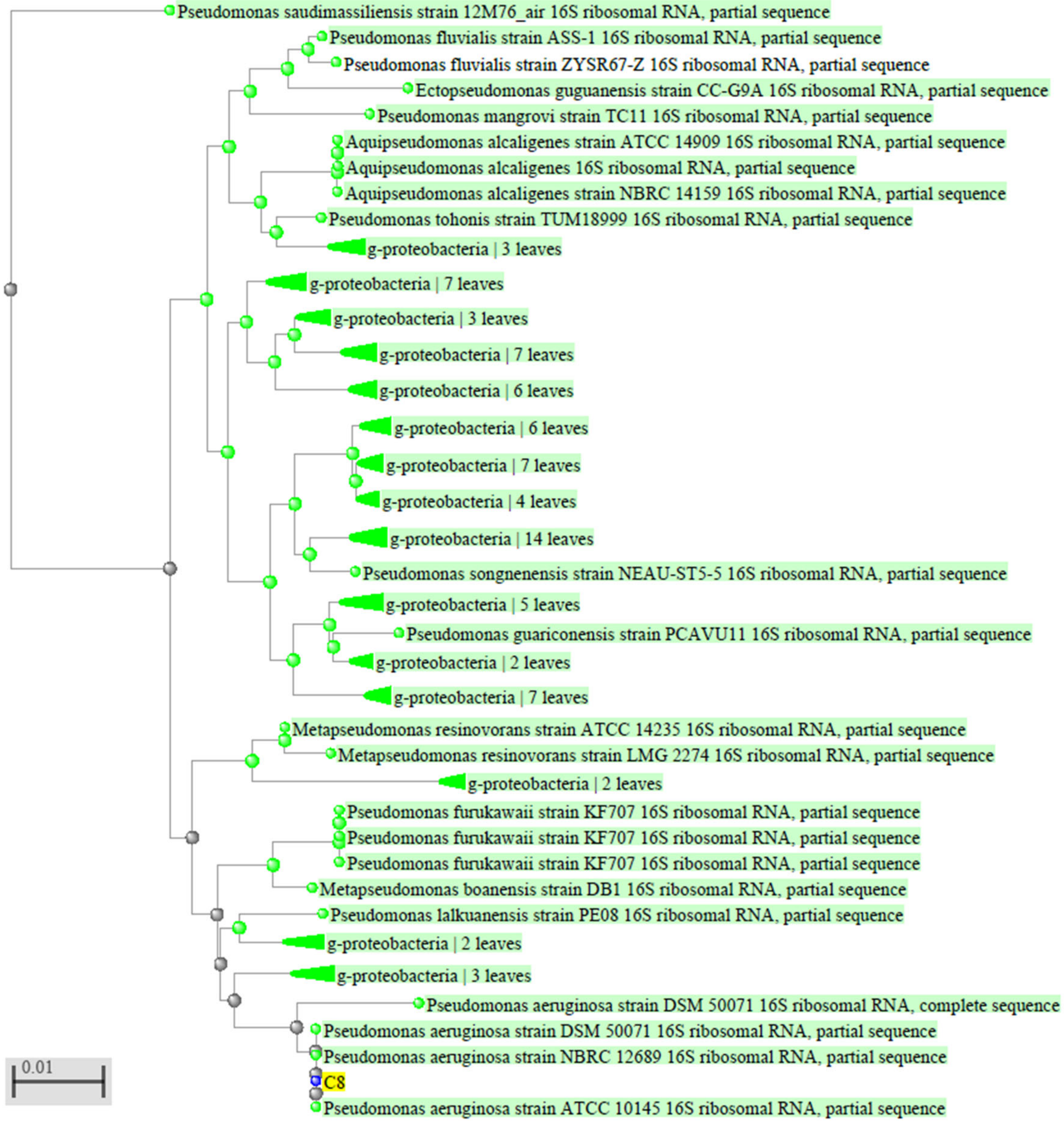

Figure S25. Phylogenetic tree of strain C8 based on 16S rRNA gene sequences showing its relationship to *Pseudomonas aeruginosa* strain DSM 50071.

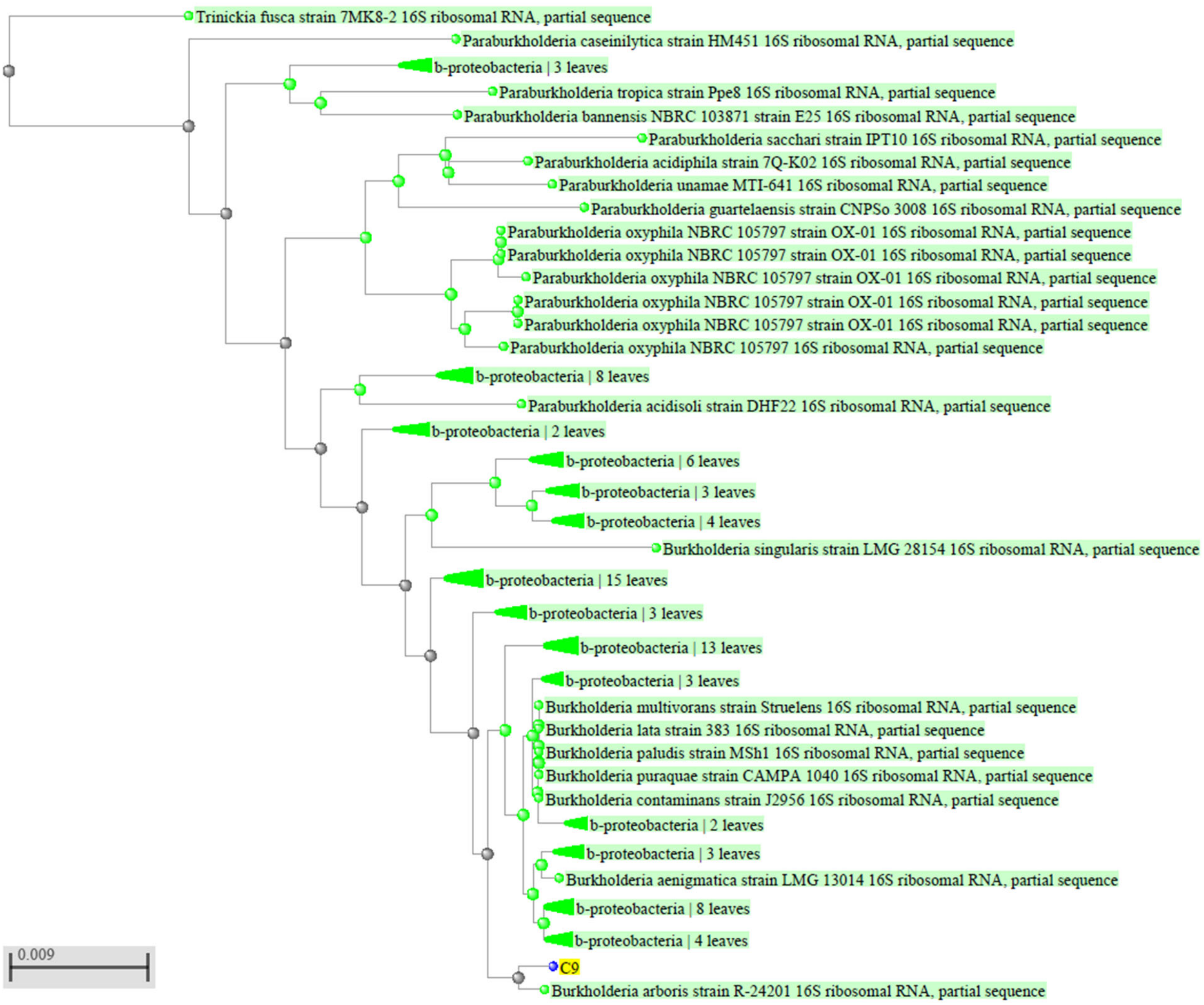

Figure S26. hylogenetic tree of strain C9 based on 16S rRNA gene sequences showing its relationship to *Burkholderia arboris* strain R-24201.

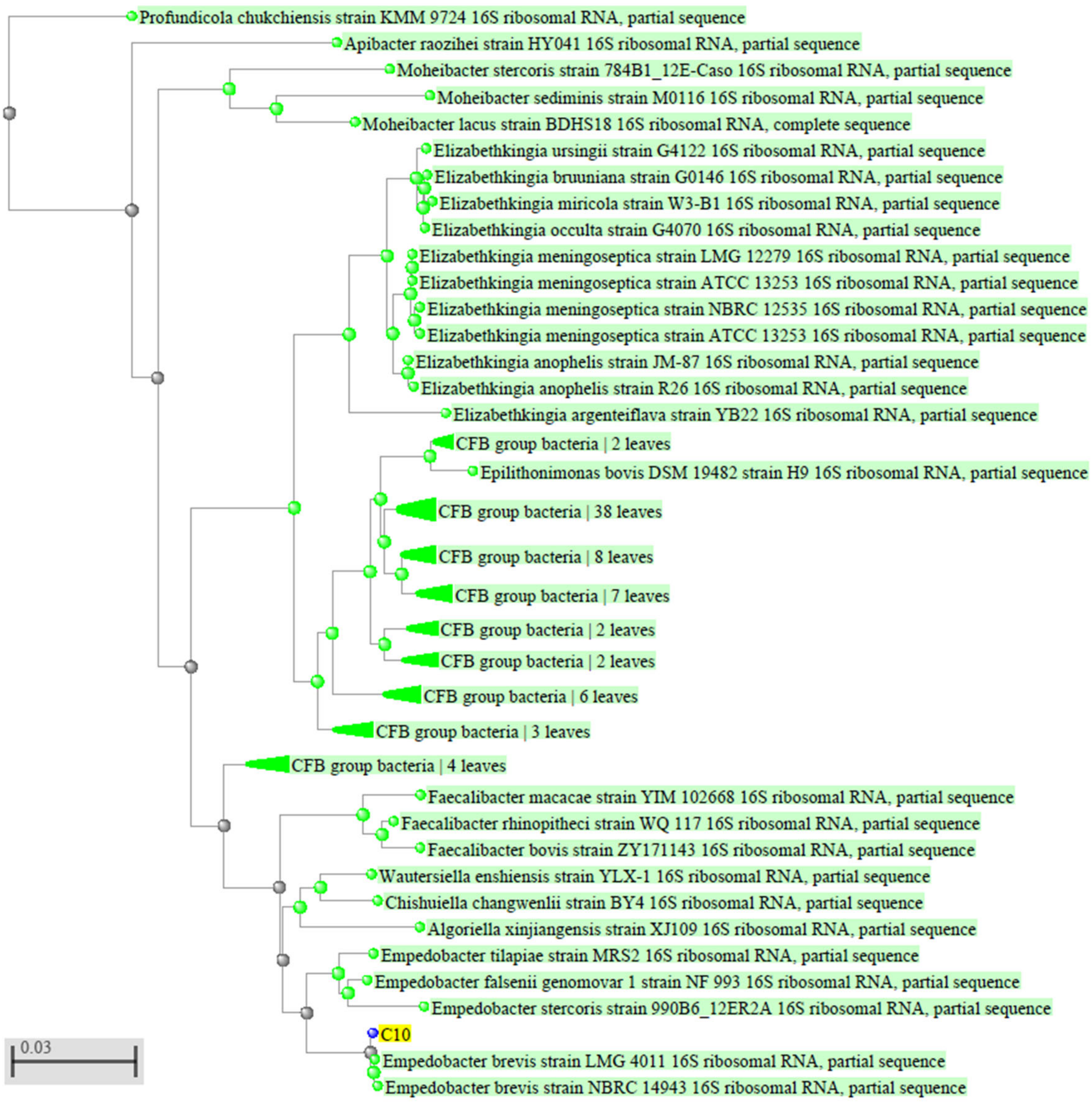

Figure S27. Phylogenetic tree of strain C10 based on 16S rRNA gene sequences showing its relationship to *Enterobacter brevis* strain LMG 4011.

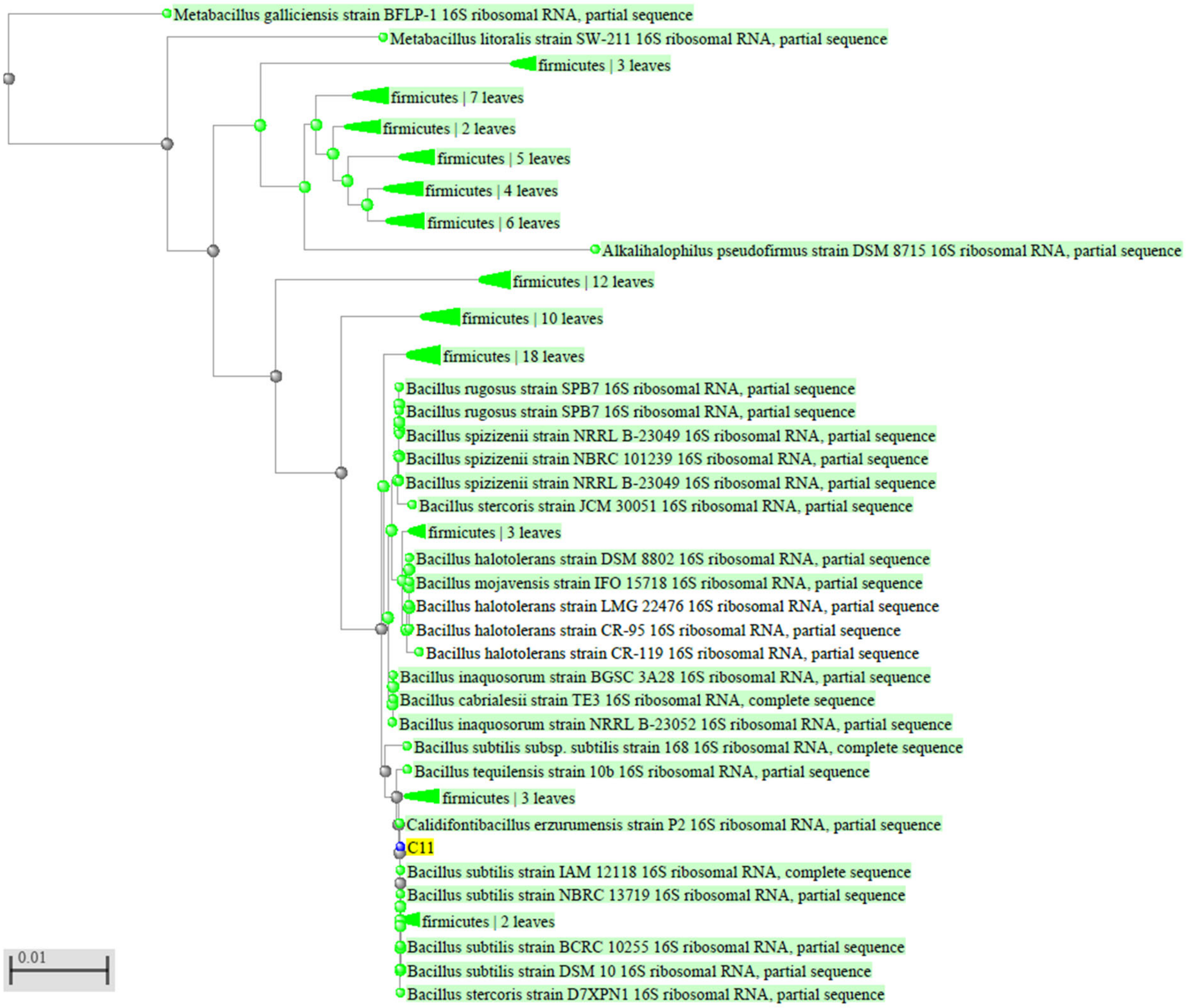

Figure S28. Phylogenetic tree of strain C11 based on 16S rRNA gene sequences showing its relationship to *Bacillus subtilis* strain IAM 12118.

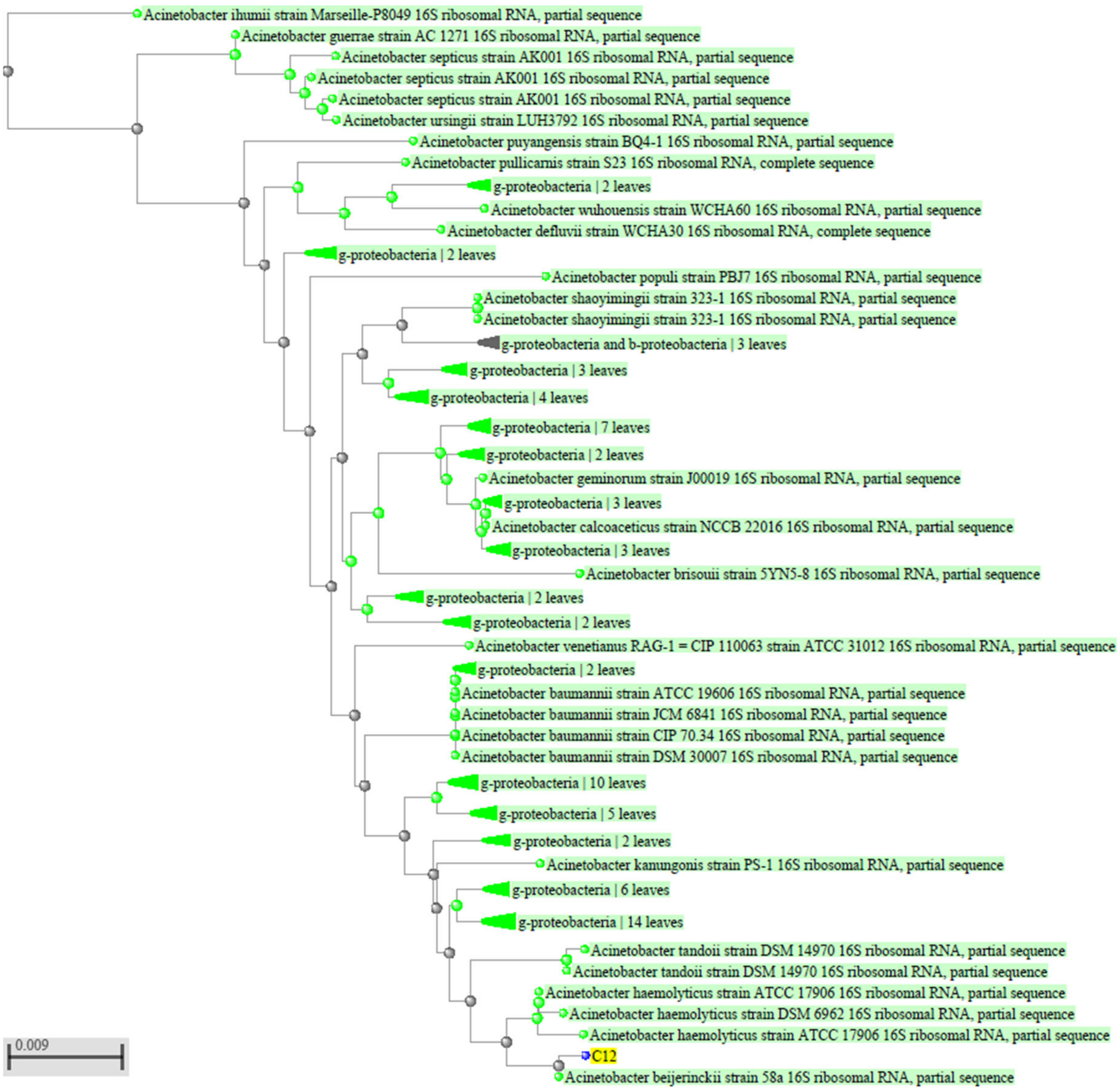

Figure S29. Phylogenetic tree of strain C12 based on 16S rRNA gene sequences showing its relationship to *Acinetobacter beijerinckii* strain 58a.

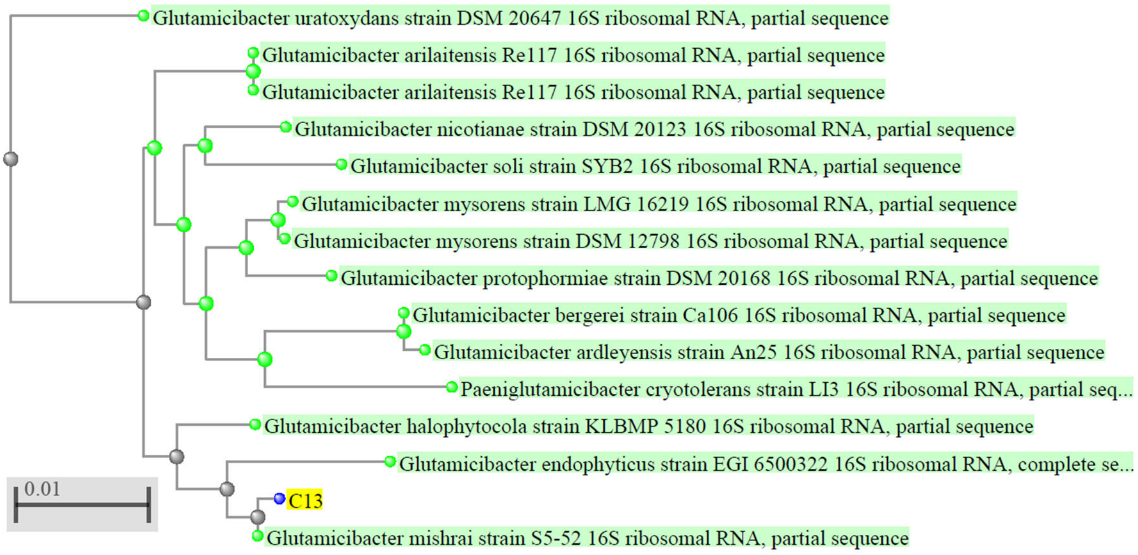

Figure S30. Phylogenetic tree of strain C13 based on 16S rRNA gene sequences showing its relationship to *Gluconobacter mishrai* strain S5-52.

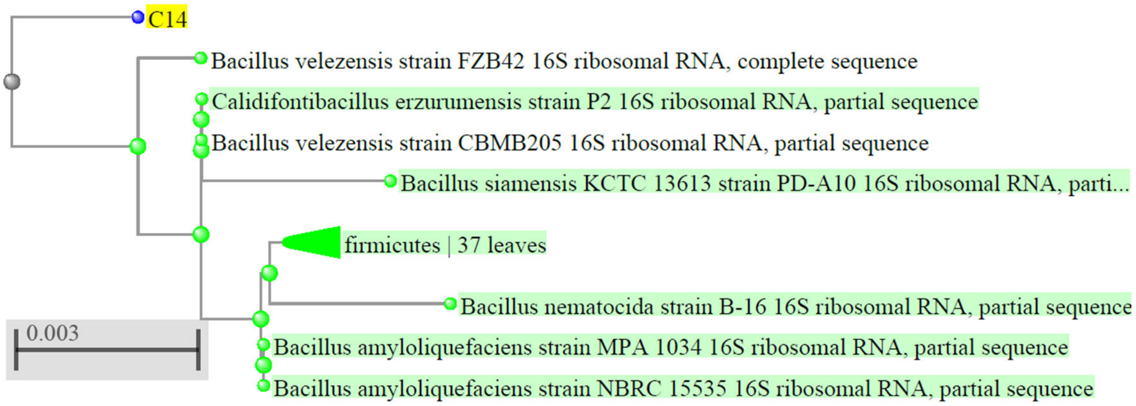

Figure S31. Phylogenetic tree of strain C14 based on 16S rRNA gene sequences showing its relationship to *Bacillus velezensis* strain FZB42.

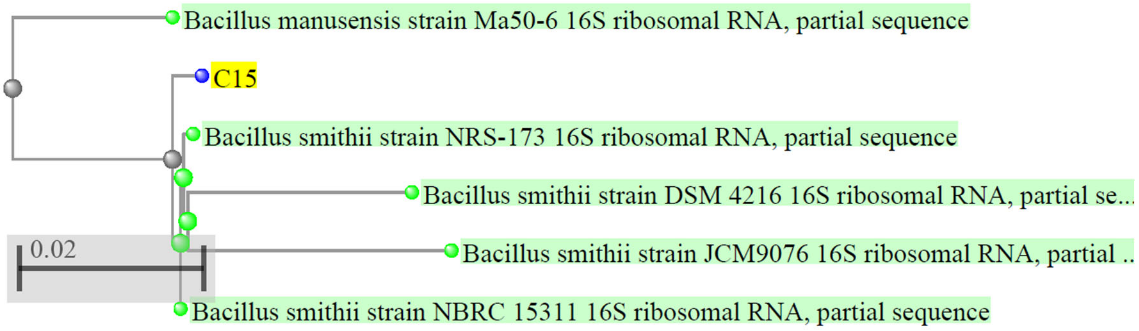

Figure S32. Phylogenetic tree of strain C15 based on 16S rRNA gene sequences showing its relationship to *Bacillus smithii* strain NBRC 15311.

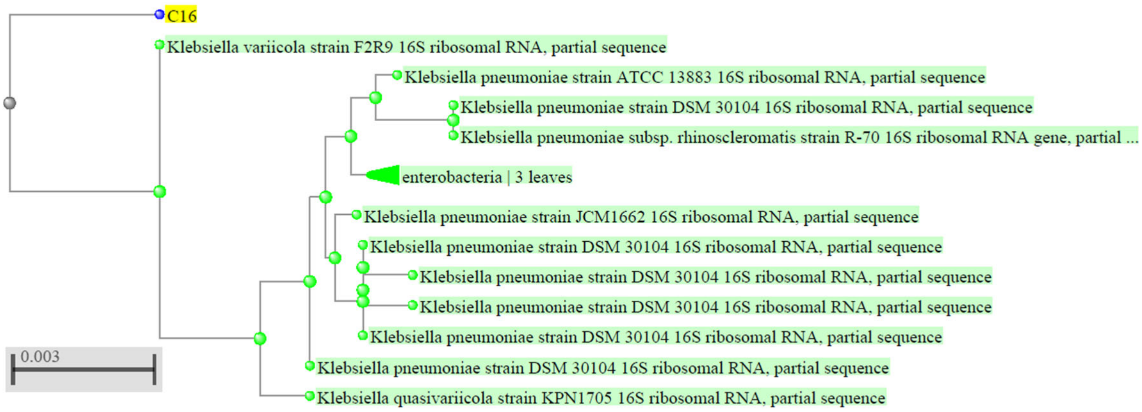

Figure S33. Phylogenetic tree of strain C16 based on 16S rRNA gene sequences showing its relationship to *Klebsiella variicola* strain F2R9.

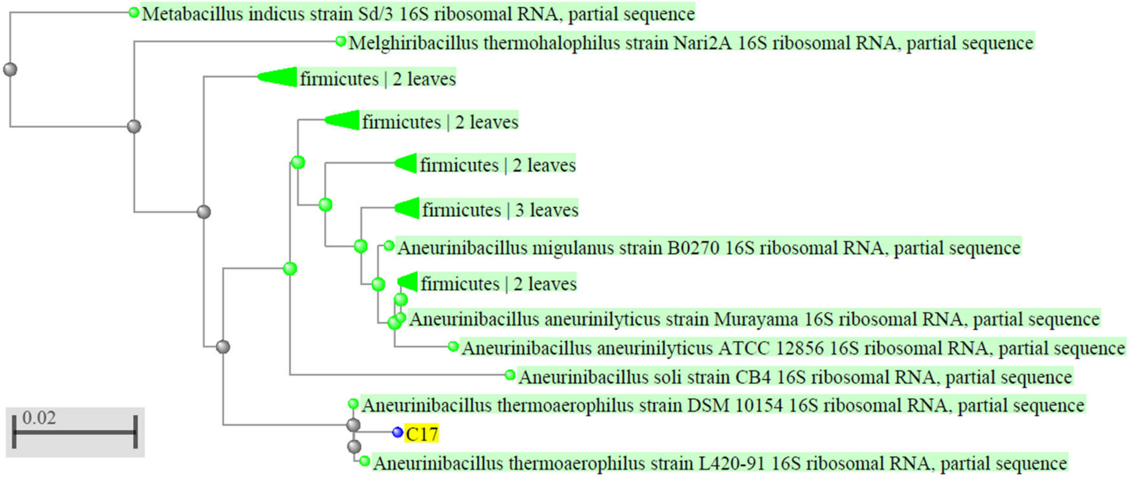

Figure S34. Phylogenetic tree of strain C17 based on 16S rRNA gene sequences showing its relationship to *Aneurinibacillus thermoaerophilus* strain DSM 10154.

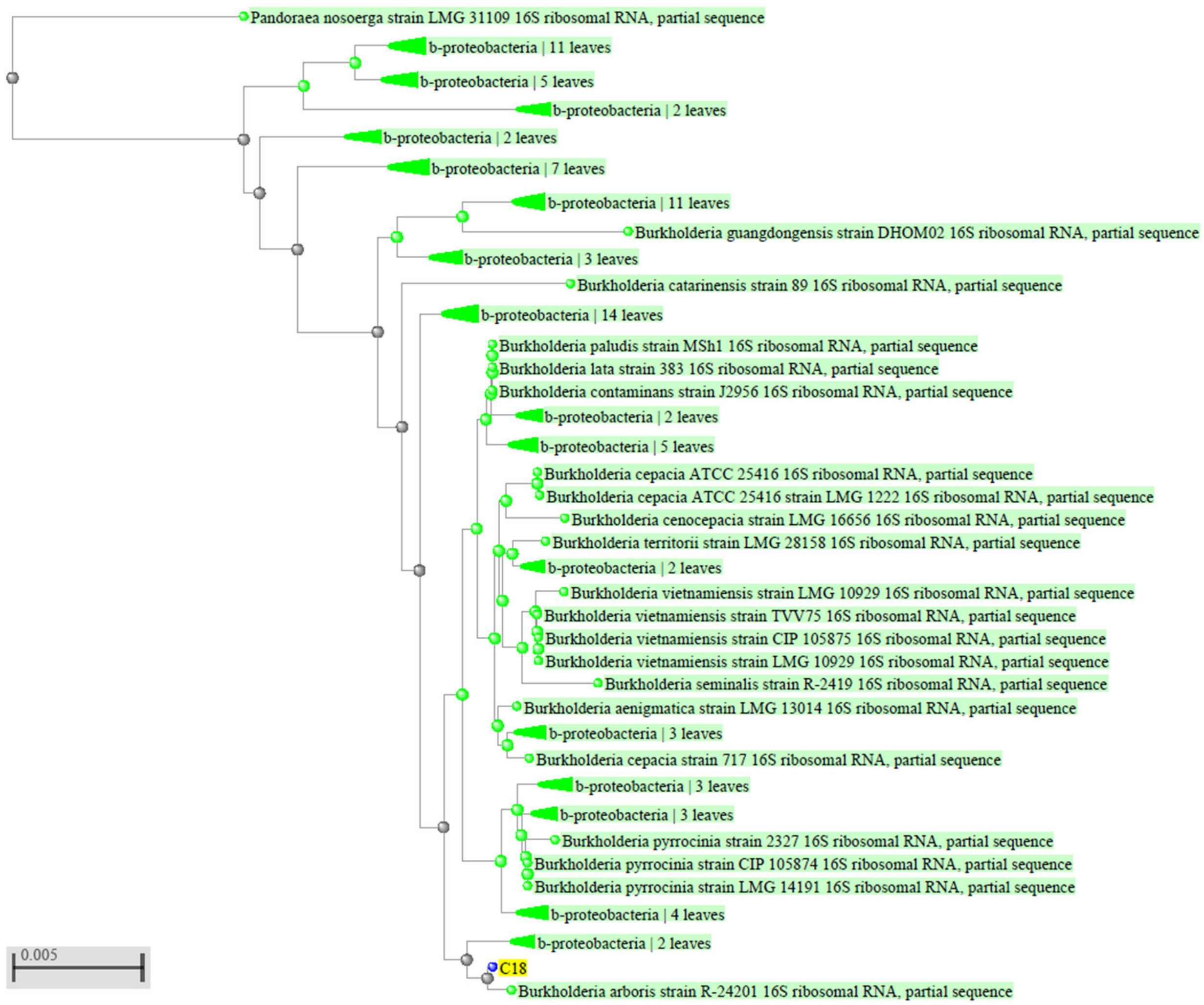

Figure S35. Phylogenetic tree of strain C18 based on 16S rRNA gene sequences showing its relationship to *Burkholderia arboris* strain R-24201.

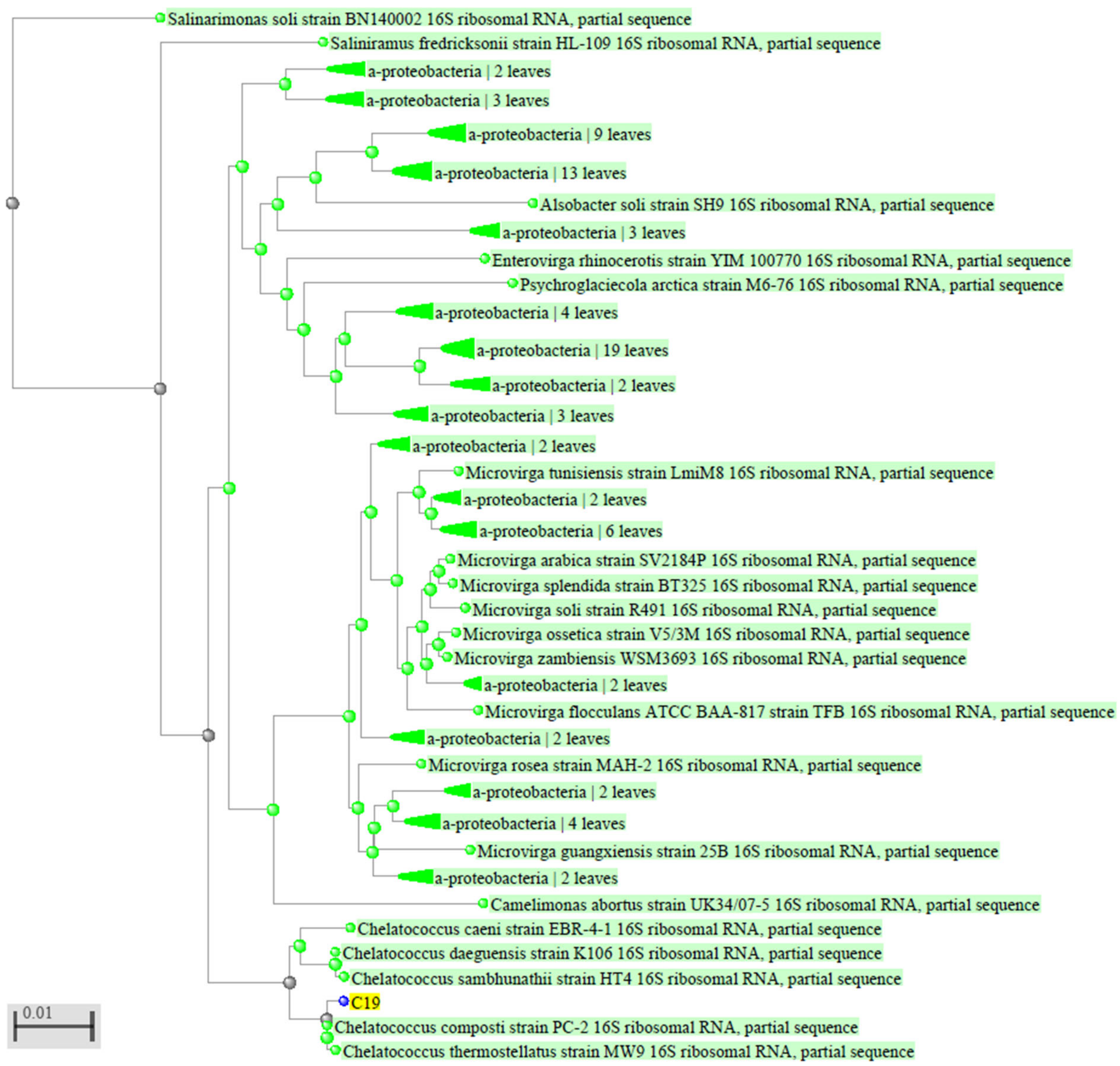

Figure S36. Phylogenetic tree of strain C19 based on 16S rRNA gene sequences showing its relationship to *Chelatococcus composti* strain PC-2.

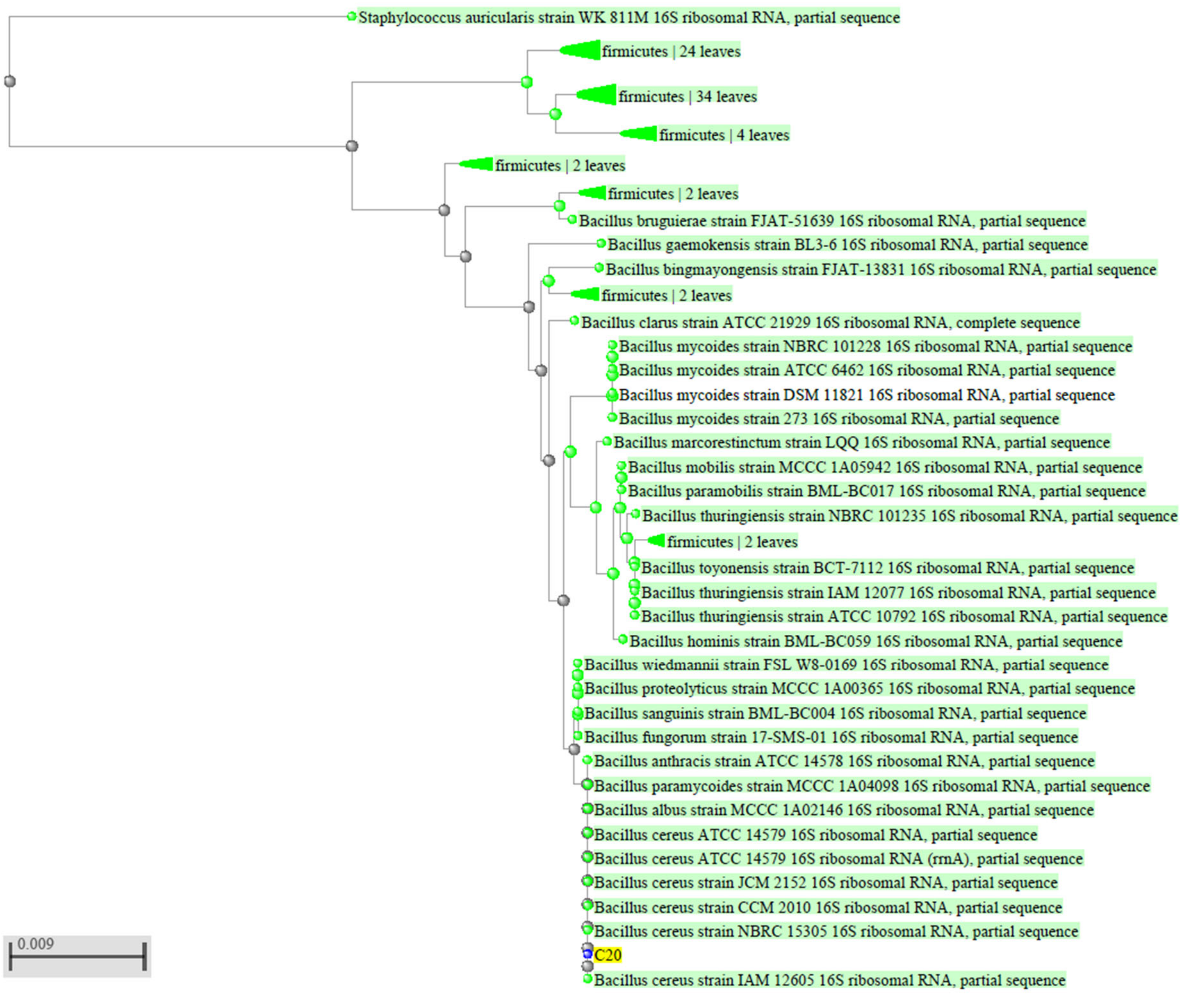

Figure S37. Phylogenetic tree of strain C20 based on 16S rRNA gene sequences showing its relationship to *Bacillus zhangzhouensis* strain MCCC 1A04098.

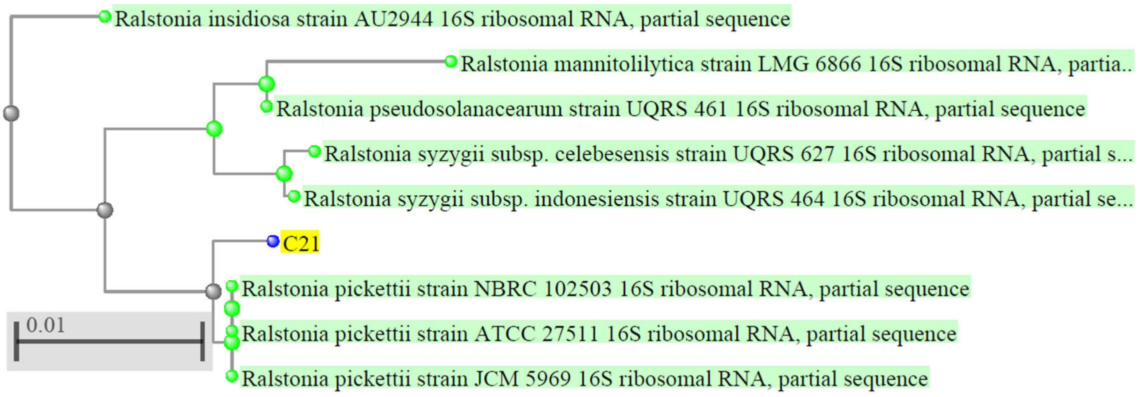

Figure S38. Phylogenetic tree of strain C21 based on 16S rRNA gene sequences showing its relationship to *Ralstonia pickettii* strain NBRC 102503.

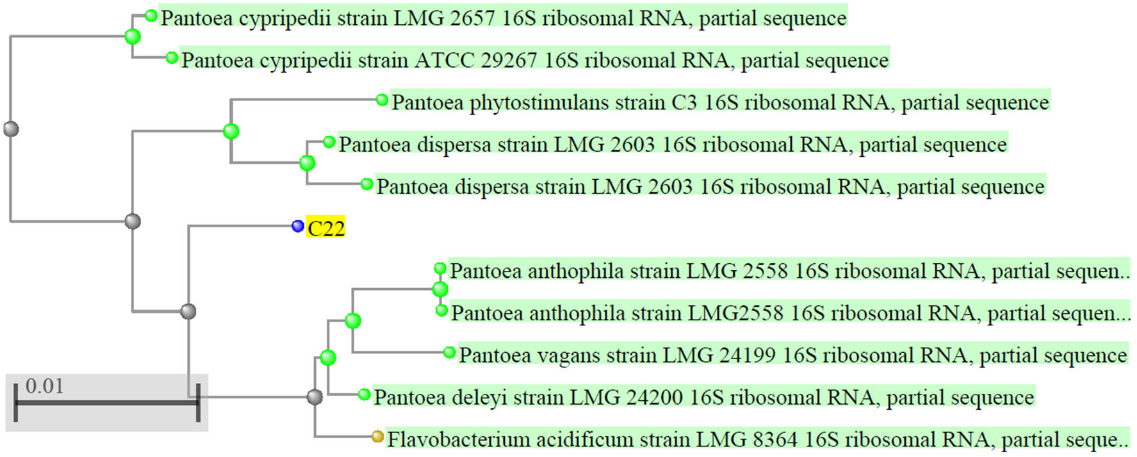

Figure S39. Phylogenetic tree of strain C22 based on 16S rRNA gene sequences showing its relationship to *Pantoea cypripedii* strain LMG 2657.

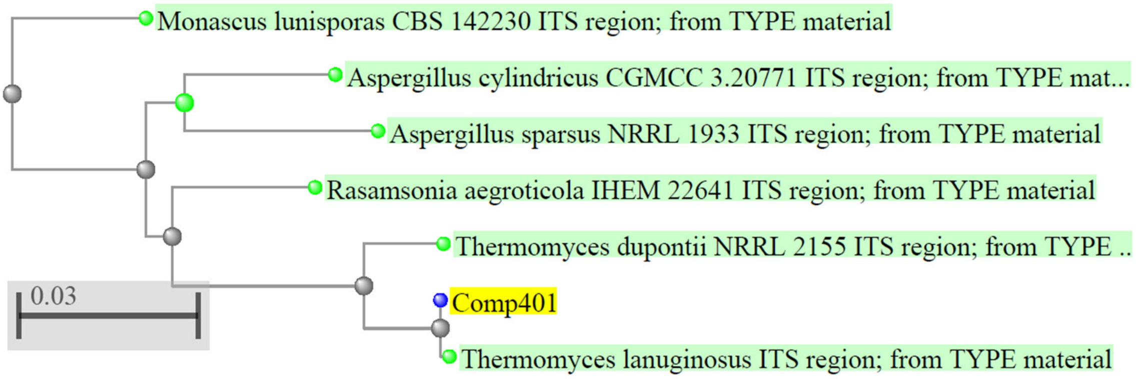

Figure S40. Phylogenetic tree of strain Comp401 based on ITS gene sequences showing its relationship to *Thermomyces lanuginosus*.

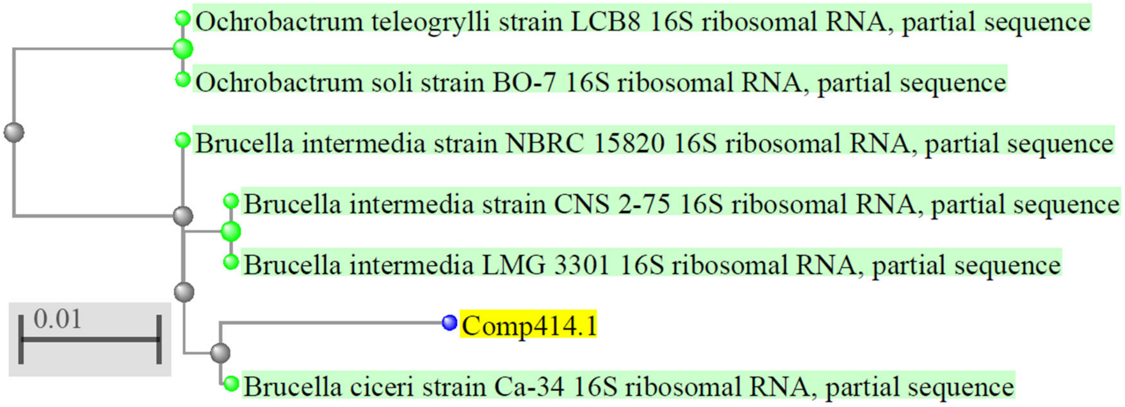

Figure S41. Phylogenetic tree of strain Comp414.1 based on 16S rRNA gene sequences showing its relationship to *Brucella ceti* strain CA-34.

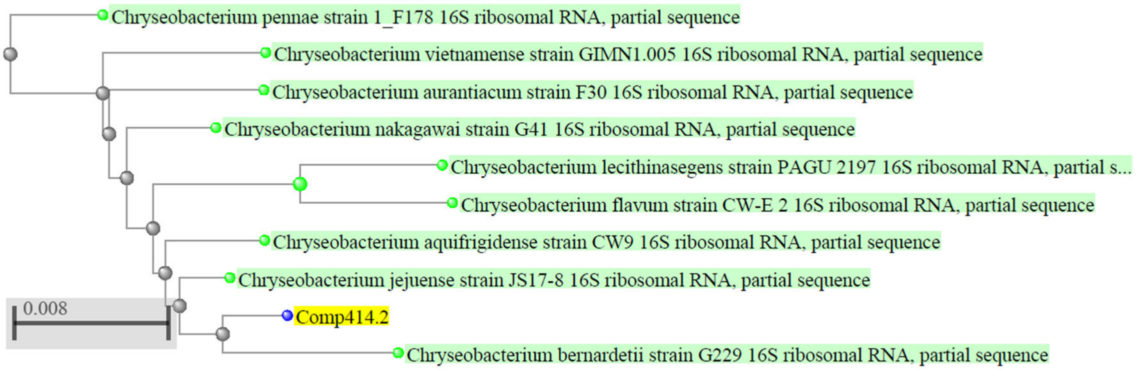

Figure S42. Phylogenetic tree of strain Comp414.2 based on 16S rRNA gene sequences showing its relationship to *Chryseobacterium jejuense* strain JS17-8.

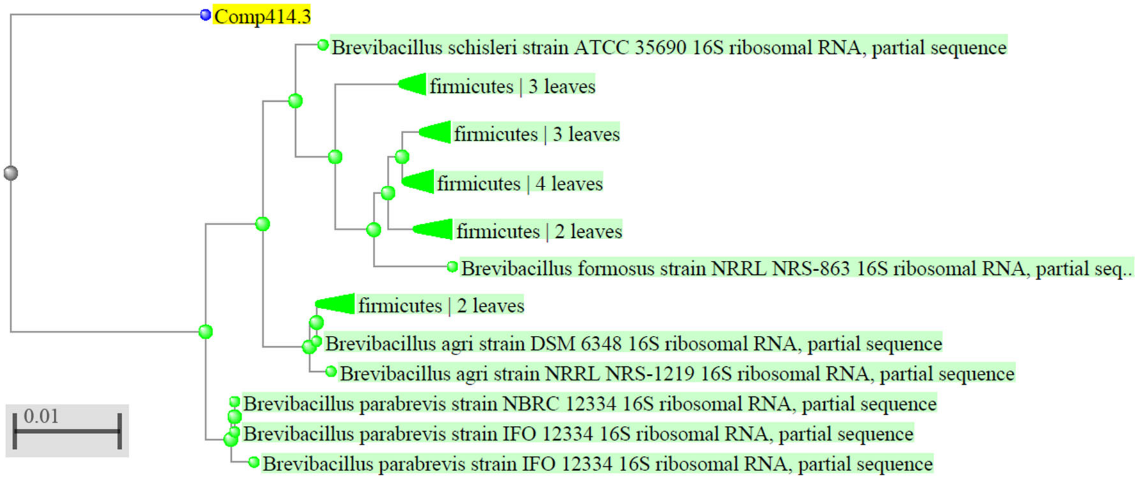

Figure S43. Phylogenetic tree of strain Comp414.3 based on 16S rRNA gene sequences showing its relationship to *Brevibacillus parabrevis* strain NBRC 12334.

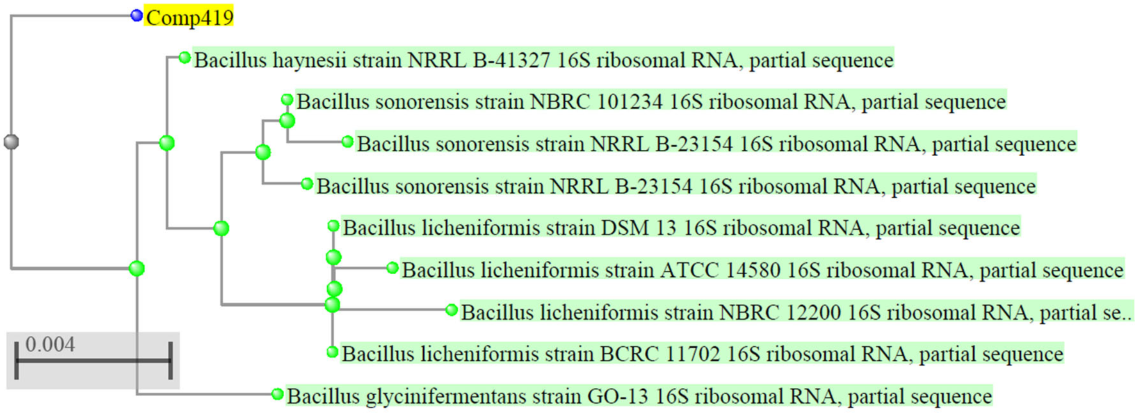

Figure S44. Phylogenetic tree of strain Comp419 based on 16S rRNA gene sequences showing its relationship to *Bacillus licheniformis* strain NRRL B-41327.

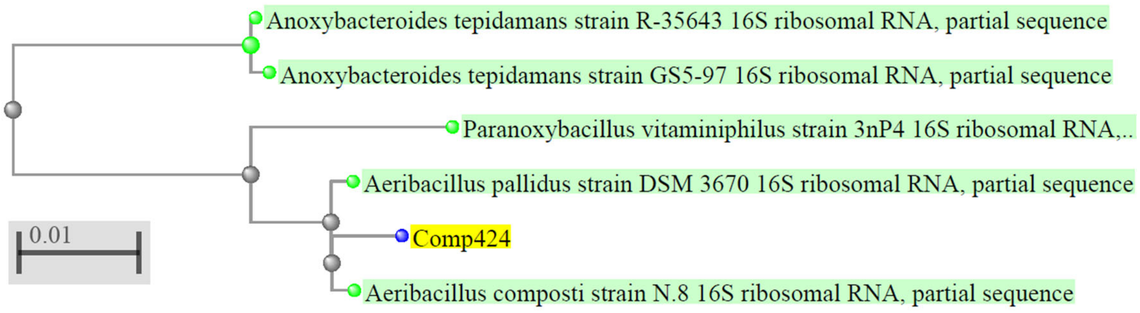

Figure S45. phylogenetic tree of strain Comp424 based on 16S rRNA gene sequences showing its relationship to *Aeribacillus* sp. strain N-8.

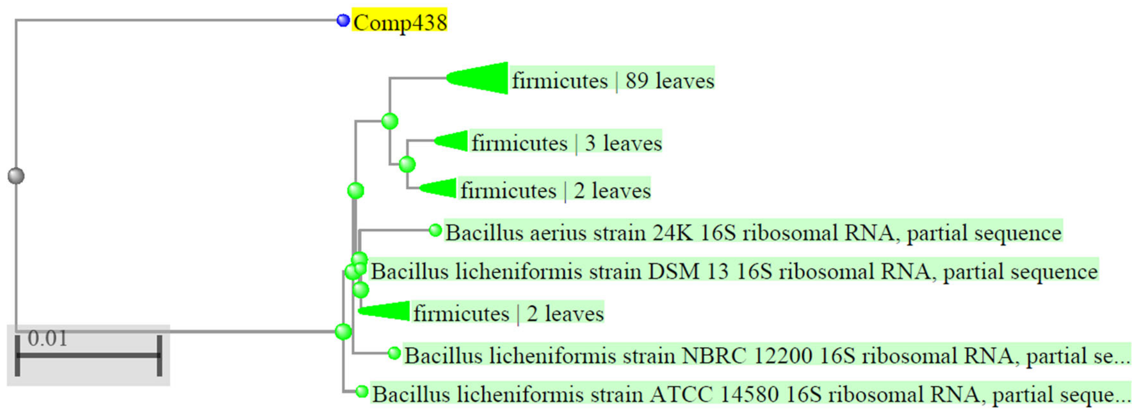

Figure S46. Phylogenetic tree of strain Comp438 based on 16S rRNA gene sequences showing its relationship to *Bacillus licheniformis* strain DSM 13.

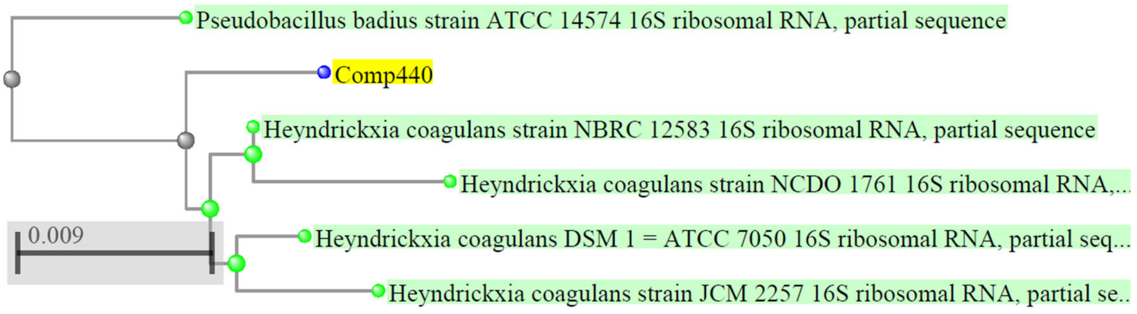

Figure S47. Phylogenetic tree of strain Comp440 based on 16S rRNA gene sequences showing its relationship to *Jeotgalibacillus coquinae* strain NBRC 12583.

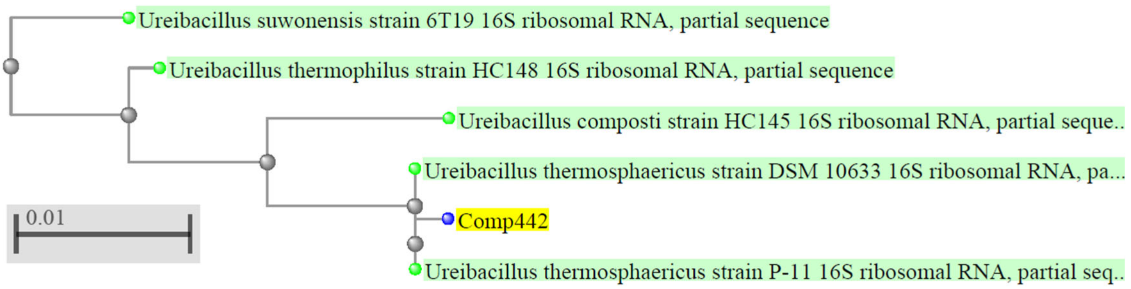

Figure S48. Phylogenetic tree of strain Comp442 based on 16S rRNA gene sequences showing its relationship to Jeotgalibacillus coquinae strain NBRC 12583.

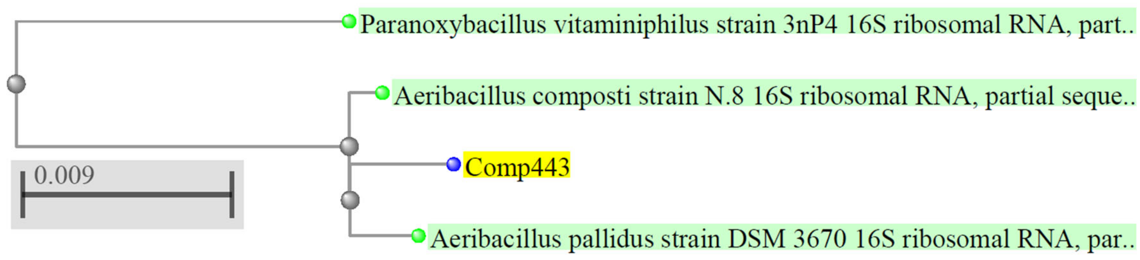

Figure S49. Phylogenetic tree of strain Comp443 based on 16S rRNA gene sequences showing its relationship to *Aeribacillus composti* strain N.8.

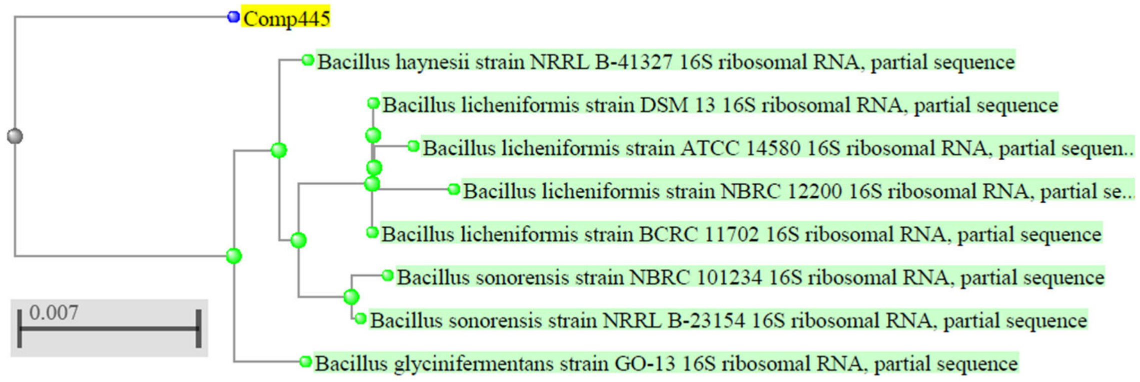

Figure S50. Phylogenetic tree of strain Comp445 based on 16S rRNA gene sequences showing its relationship to *Bacillus haynesii* strain NRRL B-41327.
